# Continuous and Scalable Production of Uniform Au Nanocrystals Capped by Citrate Species in Flow Reactors for Lateral Flow Tests

**DOI:** 10.1002/smtd.202501561

**Published:** 2025-10-21

**Authors:** Jianlong He, Kei Kwan Li, Qijia Huang, Younan Xia

**Affiliations:** ^1^ School of Chemistry and Biochemistry Georgia Institute of Technology Atlanta GA 30332 USA; ^2^ The Wallace H. Coulter Department of Biomedical Engineering Georgia Institute of Technology and Emory University Atlanta GA 30332 USA

**Keywords:** continuous flow reactor, gold nanocrystals, lateral flow test, reduction kinetics, scalable production

## Abstract

Gold (Au) nanocrystals with uniform sizes and shapes are critical for enhancing the sensitivity and reliability of lateral flow tests (LFTs). Herein, a modular platform based on flow reactors for continuous and scalable production of uniform Au nanocrystals, including clusters, small spheres, cubes, and large spheres is reported. It is demonstrated that the one‐shot injection method commonly used with batch reactors can be readily adapted for continuous flow reactors, which also allow for on‐line monitoring of growth kinetics using UV–vis spectroscopy. The uniform Au nanospheres exhibit narrower localized surface plasmon resonance peaks, greatly enhancing their mass‐to‐signal conversion ratio and diagnostic sensitivity relative to the Au nanoparticles currently used for commercial LFTs. Upon surface modification with citric acid in a flow reactor, the Au nanospheres show excellent colloidal stability, highlighting their promise for further functionalization and point‐of‐care diagnostics based on LFTs.

## Graphical abstract

A modular platform of flow reactors is developed for continuous and scalable production of uniform Au nanocrystals with different shapes. The spheres of 35 nm in diameter exhibit a narrow surface plasmon resonance peak and thus greatly enhanced color intensity, making them attractive for potential applications in high‐performance point‐of‐care diagnosis.

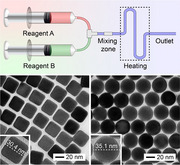

## 1 Introduction

Lateral flow tests (LFTs) have received ever‐increasing attention owing to their capability for large‐scale diagnostics and public health monitoring.^[^ ^1^, ^2^, ^3^, ^4^ ^]^ Compared to conventional molecular tests that typically involve complex laboratory procedures and prolonged waiting periods, LFTs offer rapid detection of analytes across diverse sample types with easily interpretable results.^[^ ^5^, ^6^ ^]^ These merits make them particularly well‐suited for widespread and frequent diagnosis of infectious diseases by offering advantages such as user‐friendliness, affordability, short turnaround time, and the feasibility for serial daily or weekly checking.^[^ ^7^, ^8^, ^9^ ^]^

Although LFTs were implemented on an unprecedented scale during the COVID‐19 pandemic,^[^ ^10^ ^]^ they still face a number of inherent limitations.^[^ ^11^ ^]^ For instance, conventional gold (Au) nanoparticles used in commercial LFTs often suffer from polydispersity in terms of size and shape, broadening the localized surface plasmon resonance (LSPR) peak and thereby compromising the mass‐to‐signal conversion ratio. In general, the detection sensitivity of LFTs is much lower than that of testing methods based on the reverse transcription‐polymerase chain reaction.^[^ ^12^ ^]^ The low sensitivity often leads to false‐negative results, especially during the early stage of infection. The false‐negative results can result in inadvertent high‐risk contacts and ongoing transmission.^[^ ^1^ ^]^ In addressing these issues, people are developing nanoparticle–receptor complexes through the incorporation of nanomaterials with higher sensitivity than conventional Au nanoparticles used in commercial LFTs or with new functionality to enable different detection mechanisms.^[^ ^13^ ^]^ The nanomaterials include, for example, uniform Au nanocrystals,^[^ ^14^ ^]^ latex nanoparticles,^[^ ^15^ ^]^ magnetic beads,^[^ ^16^ ^]^ nanodiamonds,^[^ ^17^ ^]^ and quantum dots^[^ ^18^ ^]^ among others.^[^ ^19^ ^]^ Among all types of uniform Au nanocrystals, Au nanospheres are expected to give the greatest sensitivity because they exhibit the narrowest LSPR peak.

In an early study, our group reported a facile method for the colloidal synthesis of uniform Au nanospheres with tunable diameters through the dropwise addition of precursor.^[^ ^20^ ^]^ However, the involvement of dropwise addition hinders the extension of such a protocol to a continuous flow reactor. As such, the Au nanospheres can hardly be produced at a volume to meet the minimum requirement for industrial evaluation. Recently, we refined the dropwise protocol by switching to one‐shot injection and successfully prepared uniform Au nanospheres with quality comparable to those synthesized using the traditional dropwise method.^[^ ^21^ ^]^ The exclusion of dropwise addition opens the door to scalable production of Au nanospheres by implementing the synthesis in the setting of a continuous flow reactor.

In this work, we successfully adapted the one‐shot injection protocol for continuous flow reactors to produce Au nanocrystals with uniform sizes and controllable shapes for high‐performance diagnostic applications. We also confirmed that the reduction kinetics of the one‐shot injection method are transferable to the flow‐reactor synthesis, facilitating its extension to large‐scale production. The setup of a flow reactor also allowed for online monitoring of the growth kinetics using UV–vis spectroscopy. When compared with the irregular Au nanoparticles used in commercial LFTs, the as‐obtained Au nanospheres exhibited narrower LSPR peaks. As a result, the nanospheres offered higher absorbance and stronger coloration even at a lower concentration to enhance the detection sensitivity. We also demonstrated that the cetyltrimethylammonium bromide/chloride (CTAB/C) on the Au nanospheres could be readily exchanged with citric acid to facilitate further functionalization with proteins needed for LFTs and other bioengineering applications.

## 2 Results and Discussion

**Figure 1:**
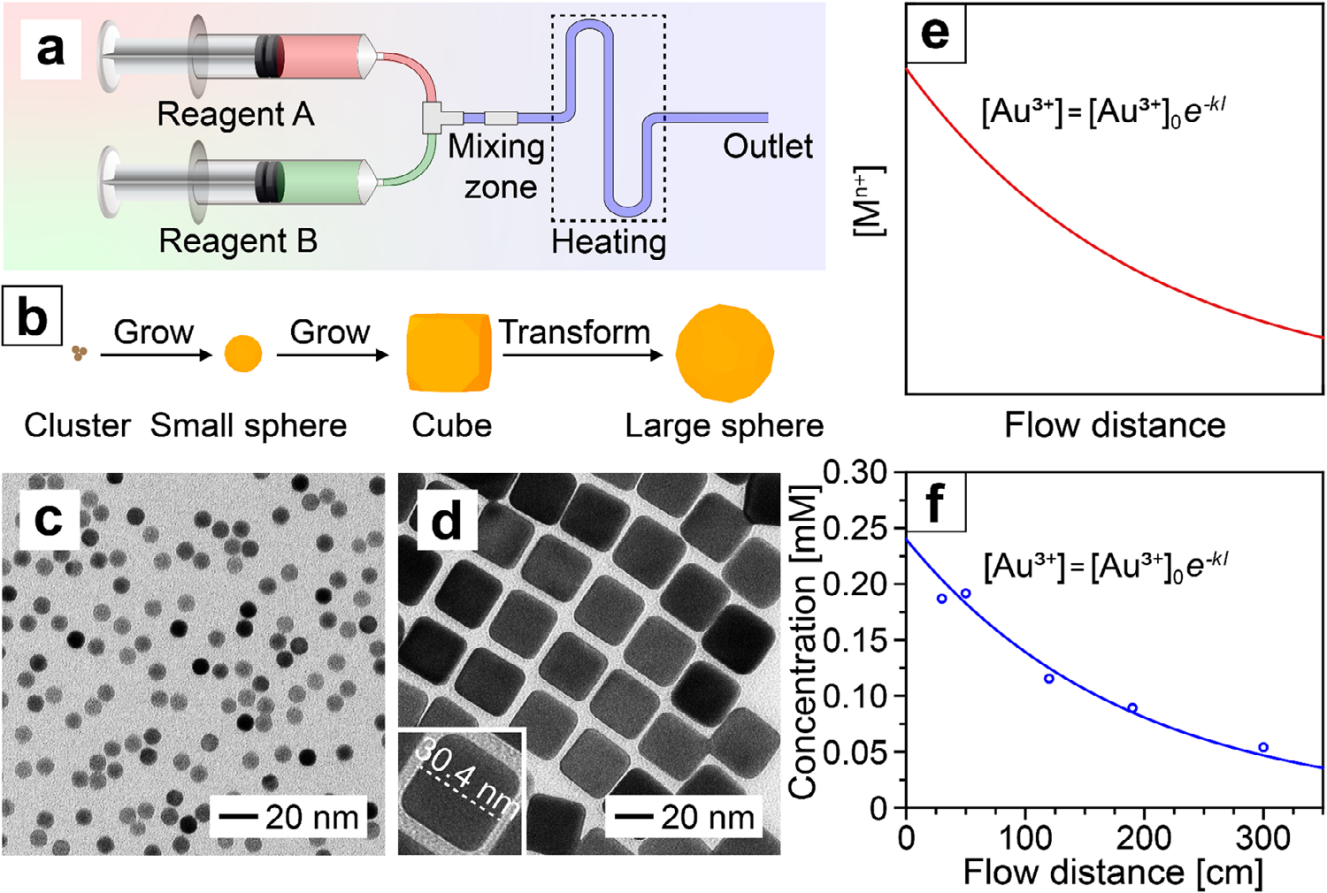
a) Schematic of the flow reactor. b) Schematic showing the growth from Au clusters to small spheres, cubes, and large spheres. c,d) Transmission electron microscopy (TEM) images of the small spheres and cubes, respectively. e) Exponential decay is expected for the remaining [Au^3+^] as a function of distance in the flow reactor. f) Experimental measurements of the remaining [Au^3+^] as a function of flow distance in a synthesis of Au cubes.

Our goal is to develop a modular flow system for the continuous and scalable production of Au nanocrystals with controllable sizes and shapes. **Figure** **1**a shows a schematic of the system. Specifically, two syringes are connected to a three‐way Luer stopcock, enabling quick mixing of solutions independently injected into the system. The solutions undergo preliminary mixing at the stopcock, followed by further homogenization in a mixing zone consisting of coupler‐adapter pairs. The two‐stage mixing ensures a uniform distribution of each reagent throughout the system (Figure S1, Supporting Information). Unlike the traditional batch reactor, where increasing the reaction volume typically results in inadequate mixing and thus non‐uniform nanoparticles (Figure S2, Supporting Information), the flow reactor can be readily adapted for the continuous and scalable synthesis of Au clusters, small spheres, cubes, and large spheres with remarkable uniformity (Figure 1b). The detailed conditions used for all the syntheses are summarized in Tables S1 and S2 (Supporting Information). First, Au clusters (Figure S3, Supporting Information) were synthesized and then directly utilized as seeds for the preparation of small Au spheres with an average diameter of 9.23 ± 0.50 nm (*n* = 212, with n being the total number of particles measured, Figure 1c; Figure S4, Supporting Information). The small spheres subsequently served as seeds for further growth into cubes with an average edge length of 30.17 ± 1.33 nm (*n* = 93, Figure 1d). It should be noted that in both the syntheses of small spheres and cubes, a high flow rate of 10 mL min^−1^, accompanied by the use of a mixing zone, was instrumental in enabling effective mixing and thus production of uniform products. Otherwise, inadequate mixing arising from a slow flow rate such as 1 mL min^−1^ and the absence of a mixing zone led to the formation of non‐uniform particles for both spheres and cubes (Figure S5, Supporting Information). In the standard synthesis, three mixers were employed, which was consistent with the setup used in the simulations. To further evaluate the effect of mixing zones, the number of mixers was systematically varied while keeping other parameters the same. For the synthesis of small spheres (Figure S6, Supporting Information), the absence of a mixer led to non‐uniform particles due to insufficient mixing of the injected solutions. Introducing a single mixer resulted in reasonably uniform nanospheres, and the uniformity was further improved by using two and three mixers. A similar trend was also observed for cubes (Figure S7, Supporting Information). Without mixer, uniform products could not be obtained. The use of one or two mixers provided partial improvement, while three or four mixers yielded highly uniform cubes. These results highlight the critical role of the mixing zone in homogenizing the growth solution and thus ensuring product uniformity.

For the seed‐mediated growth processes, they both involved two primary reactants: the Au^3+^ precursor and the reductant, which is ascorbic acid (H_2_Asc). The reaction kinetics should be governed by the second‐order rate law because the reduction requires collision and electron transfer between the precursor and reductant species involved.^[^ ^22^ ^]^ As such, the reduction rate is supposed to be directly proportional to the concentrations of the precursor and reductant.^[^ ^23^ ^]^ In the case of H_2_Asc, the carboxyl group quickly dissociates into carboxylate and proton ions, and there exists an equilibrium between H_2_Asc and HAsc^−^. Since HAsc^−^ has a stronger reducing power than H_2_Asc, it should be considered as the actual reductant that reacts with Au^3+^. Given that the concentration of HAsc^−^ remains at a more or less stable level until all the H_2_Asc has been consumed,^[^ ^24^ ^]^ the reduction can be approximated as a pseudo‐first‐order reaction with regard to the concentration of Au^3+^ ([Au^3+^]),^[^ ^25^ ^]^ see the Supporting Information for a detailed discussion. In the conventional seed‐mediated synthesis of Au nanocrystals involving a batch reactor, the precursor is introduced into the growth solution in one shot.^[^ ^21^ ^]^ As illustrated in Figure S8 (Supporting Information), upon injection, [Au^3+^] would rapidly reach a maximal value, followed by an exponential decay as a function of time. Accordingly, the reaction rate, being proportional to the concentration, is expected to exhibit a similar trend.^[^ ^26^ ^]^ In principle, this kinetic profile can also be adapted to describe the synthesis conducted in a flow reactor, with a modification to the horizontal axis from reaction time to flow distance. Figure 1e shows a plot of the expected kinetic profile, where *l* is the flow distance.

We selected the synthesis of Au cubes to analyze the reaction kinetics in the flow reactor. Specifically, we measured the mass of nanocrystals obtained at different flow distances using inductively‐coupled plasma mass spectrometry (ICP‐MS). Subsequently, we calculated the remaining [Au^3+^] at each flow distance (see the Experimental Section for more details). As shown in Figure 1f, the remaining [Au^3+^] as a function of flow distance fitted well with an exponential decay, giving a *R* ^2^ value of 0.95. The comparable reaction kinetics ensured that one could adapt one‐shot injection commonly used with a batch reactor for a flow reactor.

**Figure 2:**
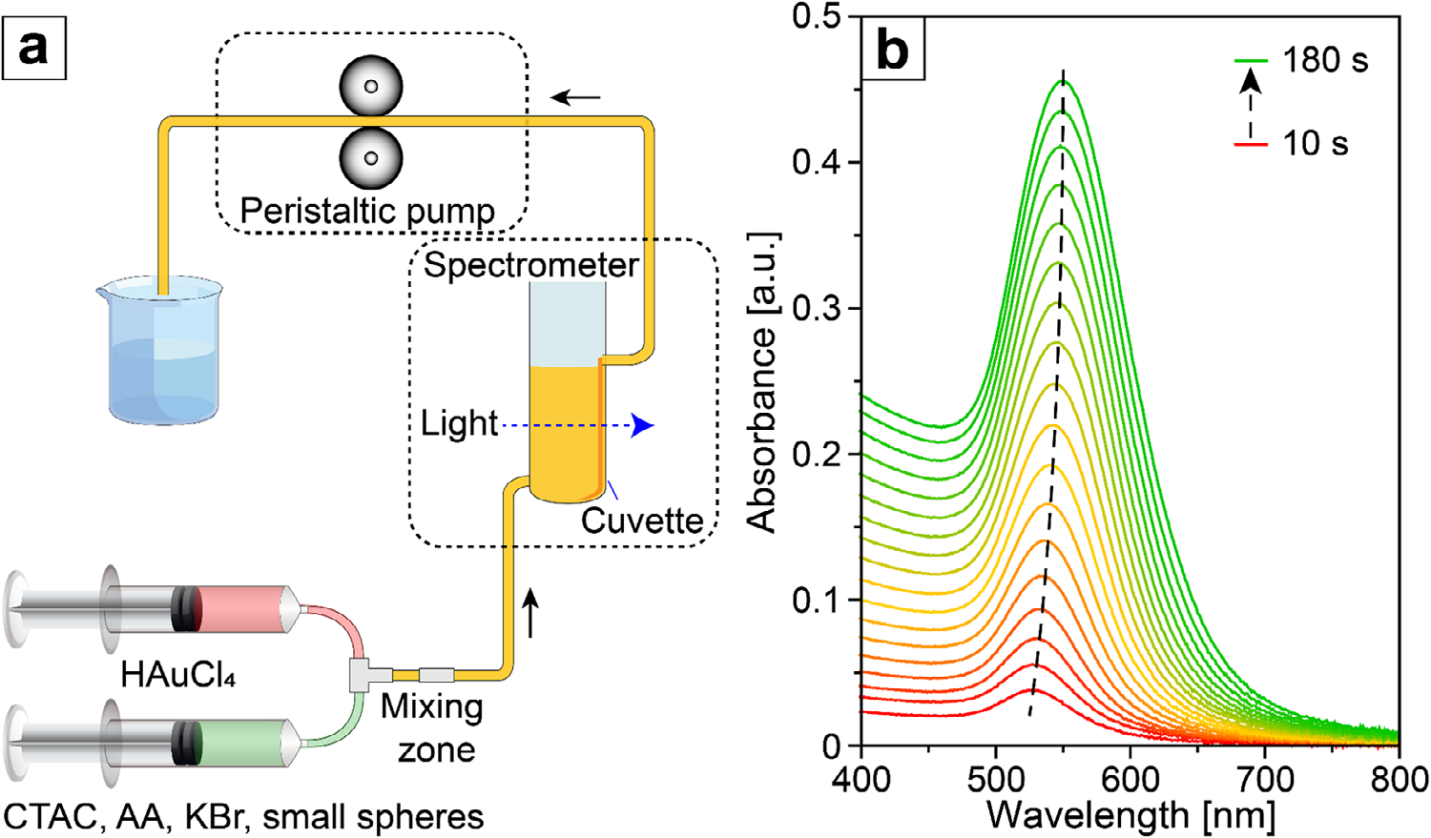
a) Schematic of the setup used for monitoring of the synthesis in situ with a UV–vis spectrometer. b) UV–vis spectra collected from the reaction solution between 10 and 180 s after mixing of the reactants during the early stage of growth from small spheres to cubes.

We then used UV–vis extinction spectroscopy to monitor the growth of small spheres into cubes in situ (**Figure** **2**a; Figure S9, Supporting Information). As the growth progressed, a gradual redshift of the LSPR peak was observed, accompanied by a continuous increase in peak intensity (Figure 2b). The redshift to LSPR peak and the increase in peak intensity can both be attributed to the increase in particle size, as well as the development of flat {100} facets and thus the appearance of sharp corners and edges.^[^ ^27^, ^28^ ^]^ Moreover, TEM analyses were performed on the samples collected at different distances along the flow direction (Figure S10, Supporting Information). The results revealed a gradual increase in particle size accompanied by a progressive shape evolution, which was consistent with the observations in the UV–vis spectra. These observations confirmed that the growth was governed by a kinetically‐controlled process that involves inadequate surface diffusion relative to deposition while demonstrating the feasibility of monitoring the growth in a flow reactor. Notably, the reaction was essentially completed within 5 min, providing useful insight for future industrial applications, where reducing reaction time can increase production efficiency.

**Figure 3:**
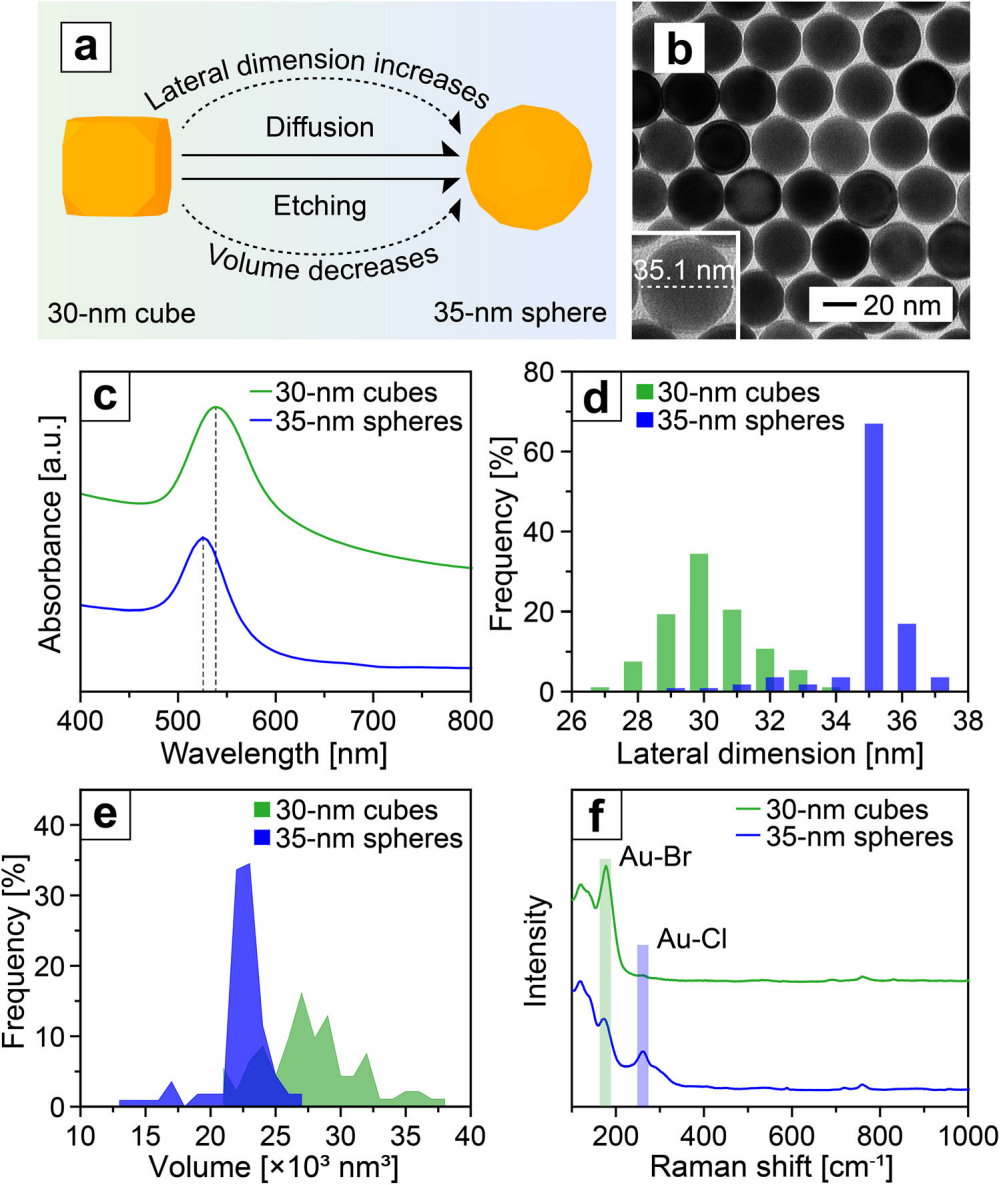
a) Schematic showing the shape transformation from a cube to a sphere. b) TEM image of the resultant 35‐nm spheres derived from the 30‐nm cubes. c) UV–vis spectra of aqueous suspensions of the 30‐nm cubes and 35‐nm spheres. d) Size distributions in terms of edge length for the cubes and diameter for the spheres. e) Volume distributions for the cubes and spheres. f) SERS spectra recorded from aqueous suspensions of the Au cubes and spheres. The color scheme applies to all panels in c–f).

Leveraging the modular nature of the flow reactor, we further extended its capability to the synthesis of large Au nanospheres. In this case, the product stream from the synthesis of Au nanocubes was directly introduced into another flow reactor without involving centrifugation or other procedures. In the second flow reactor, the cubes underwent simultaneous surface diffusion and oxidative etching processes (**Figure** **3**a), ultimately transforming them into large spheres with an average diameter of 34.96 ± 1.27 nm (*n* = 111, Figure 3b). As shown in the TEM images collected at different incubation time points (Figure S11, Supporting Information), the particles progressively evolved toward a spherical morphology with extended incubation. After 30 min, most particles appeared nearly spherical, and continued heating for 1–5 h produced uniform nanospheres. This observation indicates that prolonged heating drives the system toward the equilibrium state, leading to products with improved uniformity. The UV‐vis spectra revealed that both the cubic and spherical nanocrystals exhibited narrow LSPR peaks, suggesting good uniformity in terms of size and shape, together with excellent colloidal stability (Figure 3c).^[^ ^29^ ^]^ The peak was blue‐shifted during the shape transformation due to the truncation of corners and edges.^[^ ^30^ ^]^ The increase in lateral dimension (Figure 3d) and decrease in particle volume (Figure 3e) suggest that both surface diffusion and oxidative etching took place during the transformation from cubes to spheres. Surface‐enhanced Raman spectroscopy (SERS) analysis (Figure 3f) revealed a reduction in peak intensity for the Au–Br vibrational mode, accompanied by an increase in peak intensity for the Au–Cl vibrational mode during the transformation, suggesting the involvement of Br^−^ desorption and Cl^−^ adsorption.^[^ ^31^ ^]^ We further compared the large Au spheres with those obtained by incubating the same sample of nanocubes in a batch reactor (Figure S12a, Supporting Information). The spheres prepared in the batch reactor (average diameter = 34.70 ± 0.70 nm, *n* = 114) had a smaller diameter relative to those obtained from the flow reactor (Figure S12b, Supporting Information). The discrepancy indicated that etching played a more dominant role when the sample was incubated in a batch reactor than in a flow reactor, due to its exposure to atmosphere and thus the increased presence and supply of oxygen.

For potential industrial applications, it is also important to minimize waste and shorten reaction time. To this end, we systematically examined the effects of reactant concentration and reaction temperature while keeping other parameters fixed. For the synthesis of 9‐nm spheres (Figure S13, Supporting Information), increasing the concentrations of both the seed and precursor by threefold had almost no impact on particle uniformity, whereas fourfold increase led to a slight deviation. In the case of 30‐nm cubes (Figure S14, Supporting Information), increasing the concentrations of all reactants by twofold gave comparable results while threefold increase caused minor variation, and fourfold increase induced noticeable changes to the morphology. A similar trend was also observed regarding temperature: 9‐nm spheres remained uniform up to 50 °C, with only slight deviation at 60 °C (Figure S15, Supporting Information). However, the formation of the 30‐nm cube was more sensitive, showing a pronounced loss of uniformity at 40 °C (Figure S16, Supporting Information). These results suggest that increasing the concentrations of reactants is a practical strategy to enhance the efficiency of synthesis while reducing waste. Since both higher concentrations and temperatures accelerate the reaction kinetics, the required reaction time is correspondingly shortened, thereby reducing the residence time. However, in a continuous flow system, the effect of shortening residence time is less critical than in a batch reactor. Instead, increasing the injection rate directly improves the production throughput while ensuring sufficient mixing to preserve the product uniformity. As shown in Figure S17 (Supporting Information), the estimated yields obtained from various synthesis protocols indicate that, for 9‐nm spheres, the flow reactor delivers ≈150‐fold higher yield than the conventional batch method, while the improvement is even more pronounced for 35‐nm spheres, reaching at least 1600‐fold. Furthermore, increasing the concentrations of reactants provides an additional route to further boost the yield, underscoring the scalability of the flow reactor approach.

In practice, Au nanoparticles (≈35 nm), with a strong plasmonic resonance and an optimal size for conjugation with antibodies (≈5 nm), are commonly used as the colorant for LFTs.^[^ ^1^ ^]^ As shown in Figure S18 (Supporting Information), however, commercial LFT kits typically utilize Au nanoparticles with poorly defined sizes and shapes, resulting in a broad LSPR peak and thereby low sensitivity for visual color detection. In contrast, uniform Au nanospheres are expected to give a much sharper LSPR peak and thus higher sensitivity. As shown in the UV–vis spectra of various Au nanocrystals (Figure S19, Supporting Information), the full width at half‐maximum (FWHM) values of the LSPR peaks were determined to be 50.9 nm for the 9‐nm spheres, 80.3 nm for the 30‐nm cubes, 46.1 nm for the 35‐nm spheres, and 98.5 nm for the particles in a commercial LFT kit. These values are comparable to, or even narrower than, those reported in the literature for similarly sized Au nanocrystals, such as 56.2 nm for 10‐nm spheres,^[^ ^32^ ^]^ 68.8 nm for 10‐nm spheres,^[^ ^21^ ^]^ 85.7 nm for 10‐nm spheres,^[^ ^20^ ^]^ 68.0 nm for 30‐nm spheres,^[^ ^33^ ^]^ 48.4 nm for 35‐nm spheres,^[^ ^21^ ^]^ 68.7 nm for 40‐nm spheres,^[^ ^33^ ^]^ 51.5 nm for 46‐nm spheres,^[^ ^20^ ^]^ 59.5 nm for 50‐nm spheres,^[^ ^34^ ^]^ as well as 87.2 nm for 30‐nm cubes.^[^ ^21^ ^]^

**Figure 4:**
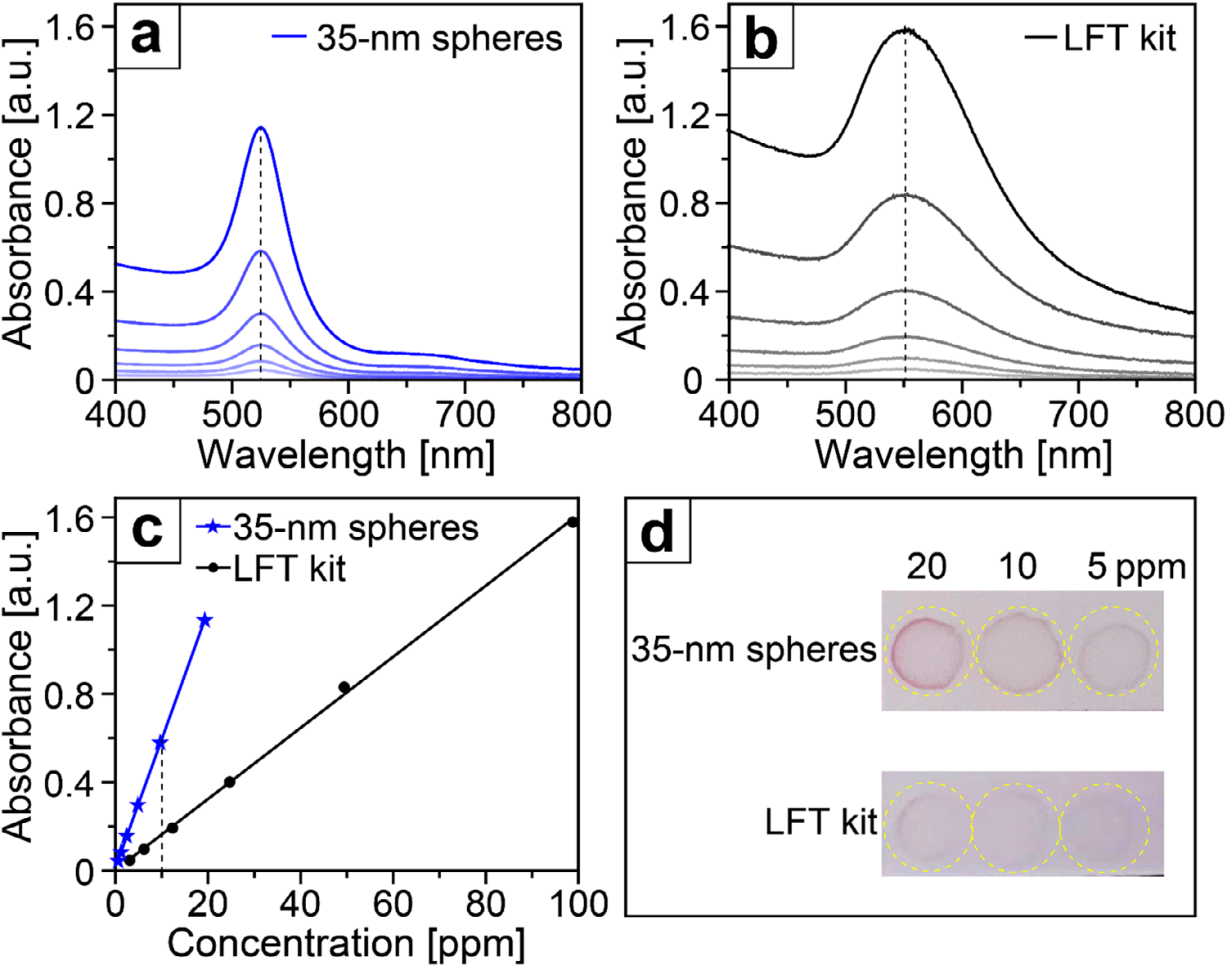
a,b) UV–vis spectra of aqueous suspensions, at decreasing concentrations of the (a) 35‐nm Au spheres and (b) Au nanoparticles harvested from a commercial LFT kit, respectively. c) Plots showing the relationship between the peak absorbance and the concentration of the two types of Au nanoparticles. d) Color appearance of the two types of Au nanoparticles at 20, 10, and 5 ppm, respectively.

**Figure** **4**a,b shows the UV–vis spectra recorded from aqueous suspensions under progressive dilution by a factor of two for the 35‐nm Au spheres and the Au nanoparticles harvested from a commercial LFT kit. As expected, the sample of 35‐nm Au spheres gave a notably narrower LSPR peak when compared to the commercial counterpart. Furthermore, the peak absorbance of the 35‐nm spheres increased linearly with concentration (Figure 4c), highlighting their potential for quantitative analysis. Significantly, at an identical atomic concentration of 10 ppm, the absorbance of the 35‐nm spheres surpassed that of the commercial counterpart by 4 times, as shown in Figure 4c. In other words, at the same concentration, the 35‐nm spheres would display a markedly stronger color (Figure 4d). Even if the concentration was reduced from 20 to 5 ppm, the pink color arising from the 35 nm Au spheres remained discernible, whereas the commercial counterpart became barely visible. Altogether, the increased efficiency in mass‐to‐signal conversion makes the uniform Au nanospheres a promising candidate for developing LFTs with improved sensitivity.

**Figure 5:**
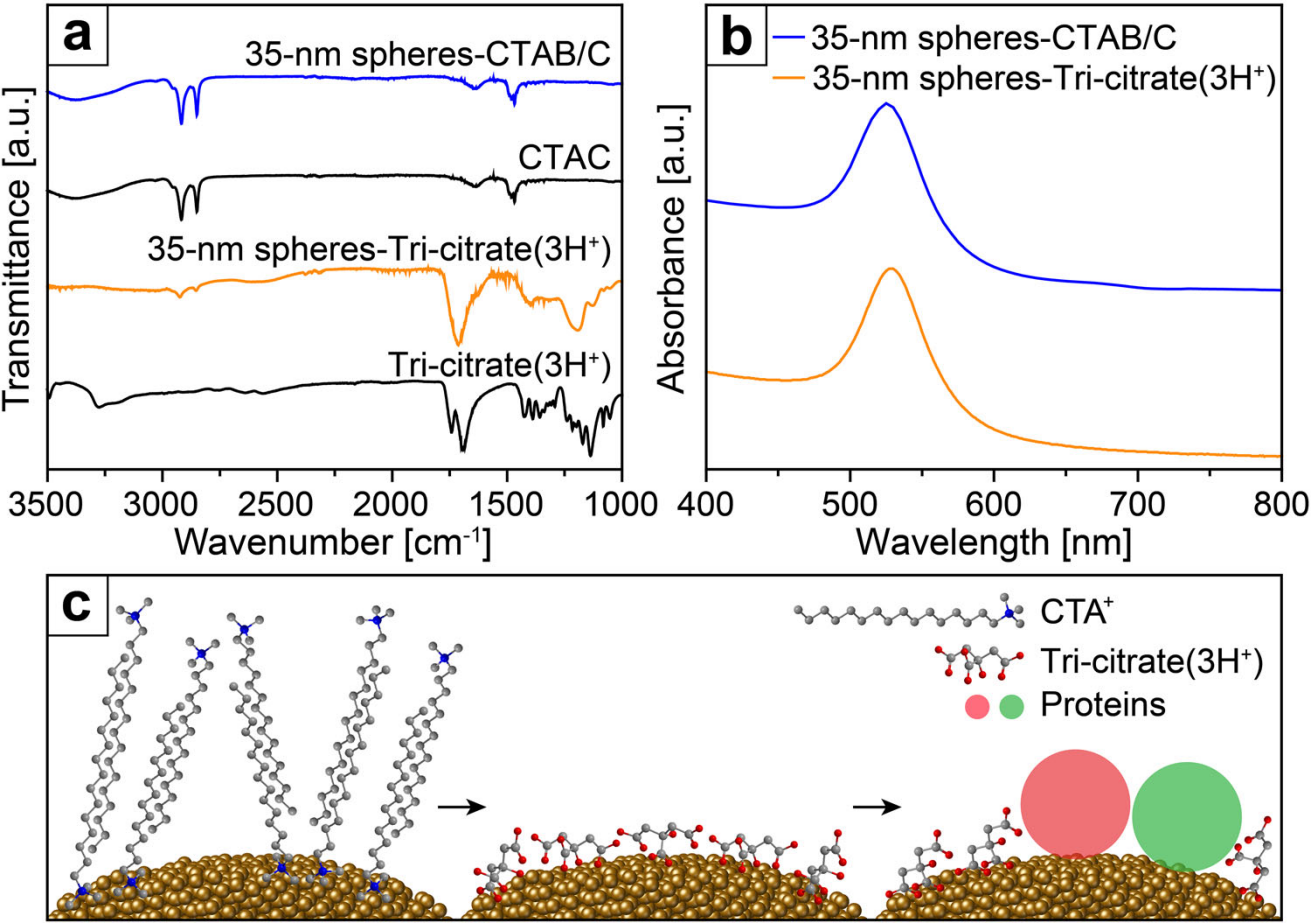
a) FT‐IR spectra of the 35‐nm Au spheres covered by CTAB/C (blue) and tri‐citrate(3H^+^) (orange), respectively. b) UV–vis spectra of the 35‐nm spheres covered by CTAB/C (blue) and tri‐citrate(3H^+^) (orange), respectively. The spectra were shifted vertically for clarity. c) Schematic illustrating the replacement of surface ligand on Au nanospheres for potential applications such as protein immobilization.

All the Au nanocrystals we synthesized were initially capped by CTAB/C, compromising their suitability for biomedical applications. Given that citrate‐stabilized Au nanoparticles are widely utilized in biomedical applications,^[^ ^35^ ^]^ we adapted the protocol developed for ligand exchange in a batch reactor to a flow reactor and successfully replaced the surface‐bound CTAB/C with citrate species to facilitate further surface modifications.^[^ ^36^ ^]^ As shown by the Fourier transform infrared (FT‐IR) spectrum in **Figure** **5**a, the as‐prepared 35‐nm Au spheres (blue traces) exhibited the characteristic doublet peaks (≈2900 cm^−1^) corresponding to the hydrocarbon chains of CTAB/C. After ligand exchange with citric acid, these peaks almost disappeared from the spectrum (orange traces), together with the appearance of peaks at 1200 and 1700 cm^−1^, which corresponded to tri‐citrate with three protons as the counterions, or tri‐citrate(3H^+^). This data confirms successful ligand exchange. Importantly, the spherical shape was well retained during the ligand exchange process (Figure S20a, Supporting Information), as well as the good colloidal stability (Figure 5b). Moreover, UV–vis analysis confirmed that the linear relationship between the peak absorbance and particle concentration remained the same after ligand exchange (Figure S20b,c, Supporting Information). Due to its relatively weak binding with the Au surface, the surface‐bound tri‐citrate(3H^+^) can be readily replaced with biological species such as peptides, proteins, and polysaccharide for a variety of biomedical applications (Figure 5c).^[^ ^37^, ^38^ ^]^

In the past decades, the most commonly used synthesis of colloidal Au nanoparticles in an aqueous system is the reduction of tetrachloroauric acid with sodium tri‐citrate, which is often referred to as “Turkevich synthesis,” named after John Turkevich who invented the synthesis in 1951.^[^ ^39^, ^40^ ^]^ These tri‐citrate‐stabilized Au nanoparticles have long been regarded as reliable starting materials for protein conjugation, and have been extensively employed in LFT applications owing to their well‐defined surface chemistry and biocompatibility.^[^ ^2^, ^41^, ^42^ ^]^ Building on this foundation, the tri‐citrate(3H^+^)‐capped Au nanospheres synthesized in flow reactors should find immediate use as a versatile platform for protein conjugation and LFT assembly. Looking ahead, it would be of great interest to further explore the direct conjugation of proteins within the same flow reactor system, streamlining the preparation process while enabling scalable production of functionalized nanoprobes for point‐of‐care diagnostics.

## 3 Conclusion

We have demonstrated the continuous production of uniform Au nanocrystals, including clusters, small spheres, cubes, and large spheres in modular flow reactors. The flow reactor allows for in situ UV–vis monitoring on the scale of second, enabling us to track the growth of small spheres into cubes, which is characterized by a gradual increase in particle size and the emergence of {100} facets and sharp corners/edges. Significantly, uniform Au nanospheres exhibit a narrow LSPR peak and thus a higher mass‐to‐signal conversion efficiency relative to the Au nanoparticles in commercial LFTs. Furthermore, the absorbance of the nanospheres also exhibits a linear correlation with the concentration, highlighting their potential for quantitative detection when integrated with digital readout devices. We have also successfully modified the surface of the nanospheres with citric acid under an acidic condition, facilitating their further functionalization with biological components. This work not only provides an effective method for the continuous and scalable production of uniform Au nanocrystals but also demonstrates their potential use to enhance LFT sensitivity, paving the way for high‐performance point‐of‐care diagnostics.

## Conflict of Interest

The authors declare no conflict of interest.

## Author Contributions

J.H. and K.K.L. contributed equally to this work. J.H., K.K.L., and Y.X. conceived the project. J.H., K.K.L., and Q.H. performed experiments and analyzed data. J.H., K.K.L., and Y.X. wrote and revised the manuscript. Y.X. provided the resources and supervised the project.

## Supporting information



Supporting Information

## Data Availability

The data that support the findings of this study are available from the corresponding author upon reasonable request.
