# Supplementary material for: Continuous and Scalable Production of Uniform Au Nanocrystals Capped by Citrate Species in Flow Reactors for Lateral Flow Tests
Source: Small Methods. 2025 Oct 21;9(12):e01561. doi: 10.1002/smtd.202501561 (PMC12716178; doi:10.1002/smtd.202501561)
Supplement: Supplementary file 1 — Supporting Information [file SMTD-9-e01561-s001.docx]

Supporting Information

**Continuous and Scalable Production of Uniform Au Nanocrystals Capped by Citrate Species in Flow Reactors for Lateral Flow Tests**

*Jianlong He, Kei Kwan Li, Qijia Huang, and Younan Xia**

J. He, K. K. Li, Q. Huang, and Prof. Y. Xia

School of Chemistry and Biochemistry, Georgia Institute of Technology, Atlanta, GA 30332, USA

E-mail: [younan.xia@bme.gatech.edu](mailto:younan.xia@bme.gatech.edu)

Prof. Y. Xia

The Wallace H. Coulter Department of Biomedical Engineering, Georgia Institute of Technology and Emory University, Atlanta, GA 30332, USA

**Chemicals, Materials, and Supplies**

Gold(III) chloride trihydrate (HAuCl_4_·3H_2_O, ≥99.9%), sodium borohydride (NaBH_4_, 98%), ascorbic acid (AA, ≥99.0%), potassium bromide (KBr, ≥99.0%), cetyltrimethylammonium bromide (CTAB, ≥99.0%), cetyltrimethylammonium chloride (CTAC, 25% in water), and citric acid were all ordered from Sigma-Aldrich and used as received. Syringes and syringe pumps were acquired from KD Scientific. Three-way stopcocks were bought from OZCZKZZ SCIENCE. Syringe adapters were obtained from AIEX. Perfluoroalkoxy (PFA) tubes were purchased from Sigma-Aldrich. COVID-19 LFT kits were obtained from Flowflex. Deionized water with a resistivity of 18.2 MΩ·cm at room temperature was used throughout the experiments.

**Characterizations**

Transmission electron microscopy (TEM) images were taken on a Hitachi HT7700 microscope operated at 120 kV. All ultraviolet-visible (UV-vis) spectra were recorded using a Cary 60 spectrometer (Agilent Technologies). The Raman spectra were captured using a Renishaw inVia Raman Spectrometer (Wotton-under-Edge, U.K.) integrated with a Leica microscope (Wetzlar, Germany). The metal contents of the samples were analyzed using an inductively-coupled plasma mass spectrometer (ICP-MS, NexION 300Q, PerkinElmer). All infrared spectra were recorded on a Shimadzu IRAffinity-1 Fourier transform infrared (FT-IR) spectrometer.

**Fabrication of the Flow Reactor**

The flow reactor was assembled from commercial components, including syringe, syringe pump, syringe adapters, three-way stopcock, and PFA tube (i.d. = 1.6 mm, o.d. = 3.2 mm, length =2 m). The reaction solution was continuously introduced into the PFA tube using syringe pumps. The reaction time was controlled by varying the flow rate and/or the length of the PFA tube. In the current work, we turned off the pump to adjust the reaction time after a certain volume of the reaction solution had been introduced into the PFA tube. This simple method allowed us to use PFA tube of 2 m in length to achieve reaction time from 15 min to 5 h in a proof-of-concept demonstration.

**Analysis of Reduction Kinetics**

In a typical colloidal synthesis of Au nanoparticles, when H_2_Asc is used as the reductant, the carboxyl group quickly dissociates into carboxylate and proton ions. There exists an equilibrium between H_2_Asc and HAsc^-^, while HAsc^-^ can be subsequently oxidized to dehydroascorbic acid (DHA) by reacting with the metal ion:

$H_{2}\mathrm{Asc}\underset{k_{-1}}{\underset{\rightleftharpoons}{k_{1}}}\mathrm{HAsc}^{-}\underset{\to}{k_{2}}\mathrm{DHA}$, (1)

As a result, HAsc^-^ should serve as the actual reductant to react with Au^3+^. Regardless of the concentration of H_2_Asc (added into the reaction system in one shot) in the reaction mixture, the concentration of HAsc^-^ is determined by the dissociation constant (*K_a_*) of H_2_Asc and the pH: [HAsc^-^] = *K_a_*/[H^+^]. The constant reaction temperature and relatively low concentration of H_2_Asc would keep *K_a_* and [H^+^] at constant levels, respectively. As such, the actual reductant, HAsc^-^, can be considered to remain at a constant concentration during the synthesis. Under this assumption, the reduction can be approximated as a pseudo-first-order reaction with regard to the concentration of Au^3+^ ([Au^3+^]):

*R* = –d[Au^3+^]/d*t* = *k*·[Au^3+^], (2)

where *k* is the rate constant, whose magnitude depends on the coordination ligand binding to the metal ion, the type and concentration of the reductant, as well as the number of active sites on the surface of the growing nanocrystals (or preformed seeds added into the reaction mixture).

When seeds are present in the growth solution, the precursor can be reduced in the solution and at active sites located on the seed surface. Taken together, the reduction of Au^3+^ may follow two different pathways: reduction in the solution phase (Au^3+^ + 3e^-^ → Au, with a rate constant of *k’*) or autocatalytic reduction on the surface of the seeds (Au^3+^ + Au_s_ + 3e^-^ →Au_s+1_, with a rate constant of *k’’*). The overall reduction rate can be expressed as:

*R* = *k’* [Au^3+^] + *k’’* [Au^3+^][Au_s_], (3)

where [Au_s_] is the concentration of active sites located on the surface of the seeds. In principle, the rate constants *k’* and *k’’*, corresponding to solution and surface reduction, respectively, can be obtained by ﬁtting the curve obtained by plotting the precursor concentration as a function of reaction time. Due to the lack of in situ characterization tools that can directly observe the growth of nanocrystals with satisfying spatial and temporal resolutions, it is still impossible to calculate [Au_s_] on the growing seeds at different stages of a synthesis. As a good approximation, it can be assumed that the concentration of active sites located on the seeds remains constant. This assumption will introduce a certain error, but the error should be within the acceptable range because reduction tends to occur at active sites that are greatest in surface energy, rather than over the entire surface. Under this assumption, the overall reduction rate becomes:

*R* = *k* [Au^3+^], *k* = *k’* + *k’’*[Au_s_], (4)

Upon integration, we obtain:

[Au^3+^] = [Au^3+^]_0_·*e^–kt^*, (5)

with [Au^3+^]_0_ being the initial concentration of Au^3+^ in the reaction solution. The instantaneous reduction rate can be written as:

*R* = *k* [Au^3+^]_0_·*e^–kt^*. (6)

In the conventional seed-mediated synthesis of Au nanocrystals involving a batch reactor, the precursor is introduced into the growth solution in one shot. As illustrated in Figure S6, upon injection, both the [Au^3+^] (Equation 5) and reduction rate (Equation 6) would rapidly reach maximal values, followed by exponential decays as a function of time. In principle, the kinetic profile can also be adapted to describe the synthesis conducted in a flow reactor by simply switching the horizontal axis from reaction time to flow distance:

[Au^3+^] = [Au^3+^]_0_·*e^–kl^*, (7)

where *l* is the flow distance.

**Measurement of Reduction Kinetics in a Flow Reactor**

First, we prepared a set of flow reactors with PFA tubes of varying lengths. Meanwhile, 1.5 mL centrifuge tubes containing 500 µL of ethanol were pre-cooled at –80 °C for 3 h. The growth solution was identical to what was used in a typical synthesis of Au nanocubes using 9-nm Au nanospheres as the seeds. At the outlet of each PFA tube of varying lengths, 500 µL of the growth solution was collected and immediately injected into the pre-cooled ethanol hosted in a centrifuge tube. The tube was then quickly placed in a –20 °C freezer. Afterwards, the sample was centrifuged at 17000 rpm and –10 °C for 30 min. The supernatant was discarded, and the pellet was collected for ICP-MS analysis to determine the amount of precursor that had been reduced to the elemental form.

**Monitoring the Growth of Small Spheres into Cubes in a Flow Reactor**

First, we prepared a set of flow reactors with PFA tubes of varying lengths, corresponding to different residence time. Meanwhile, 1.5 mL centrifuge tubes containing 800 µL of ethanol were pre-cooled at –80 °C for 3 h. The growth solution was identical to what was used in a typical synthesis of Au nanocubes using 9-nm Au nanospheres as the seeds. At the outlet of each PFA tube of varying lengths, 300 µL of the growth solution was collected and immediately injected into the pre-cooled ethanol hosted in a centrifuge tube. The tube was then quickly placed in a –20 °C freezer. Afterwards, the sample was centrifuged at 14500 rpm and –10 °C for 30 min. The supernatant was discarded, and the pellet was collected for TEM analysis.

**Monitoring the Evolution of Cubes into Large Spheres in a Flow Reactor**

First, we prepared a set of flow reactors with PFA tubes of varying lengths, corresponding to different residence time. Meanwhile, 1.5 mL centrifuge tubes containing 800 µL of ethanol were pre-cooled at –80 °C for 3 h. The incubation solution was identical to what was used in a typical incubation process. At the outlet of each PFA tube of varying lengths, 300 µL of the incubation solution was collected and immediately injected into the pre-cooled ethanol hosted in a centrifuge tube. The tube was then quickly placed in a –20 °C freezer. Afterwards, the sample was centrifuged at 14500 rpm and –10 °C for 10 min. The supernatant was discarded, and the pellet was collected for TEM analysis.

**Ligand Exchange in a Flow Reactor**

The 35-nm nanospheres dispersed in water at a concentration of 0.05 mg mL^-1^ were used as reagent A and an aqueous solution of citric acid at 1 M was used as reagent B. Reagents A and B were injected into the flow reactor at the same flow rate of 10 mL min^-1^, followed by incubation at 95 °C for 5 h. Finally, the solid sample was collected by centrifugation at 15000 rpm for 10 min and washed twice with water.


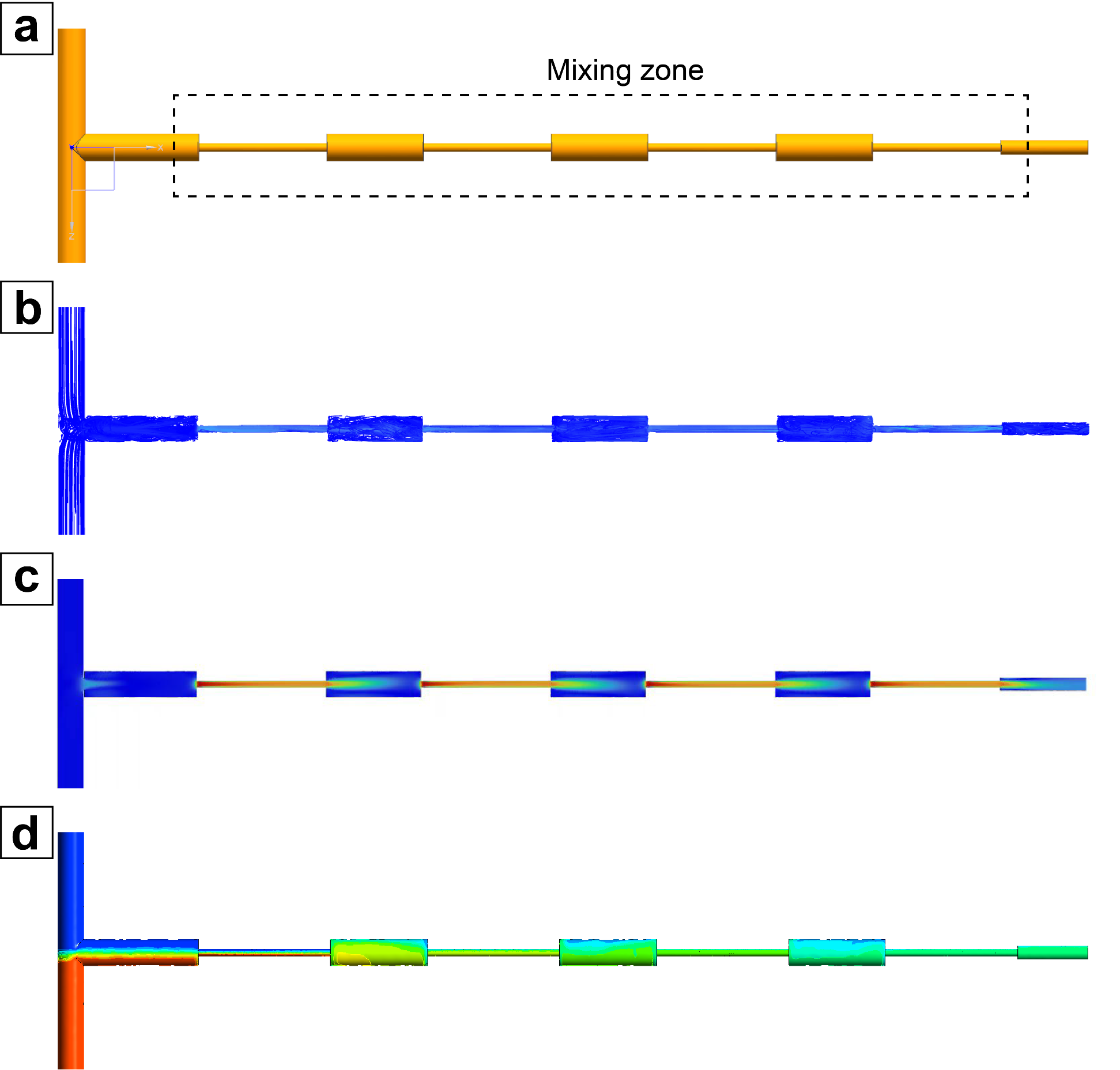


**Figure S1.** (a) Three-dimensional model of a typical flow reactor channel. (b) Simulated distribution of liquid flow in the channel. (c) Simulated distribution of flow velocity, with the color gradient from blue to red indicating increasing velocity. (d) Simulated visualization of the mixing between two liquid streams that were initially labeled in blue and red, respectively. In (b-d), a flow rate of 10 mL min⁻¹ was assigned to both liquids in the simulation.


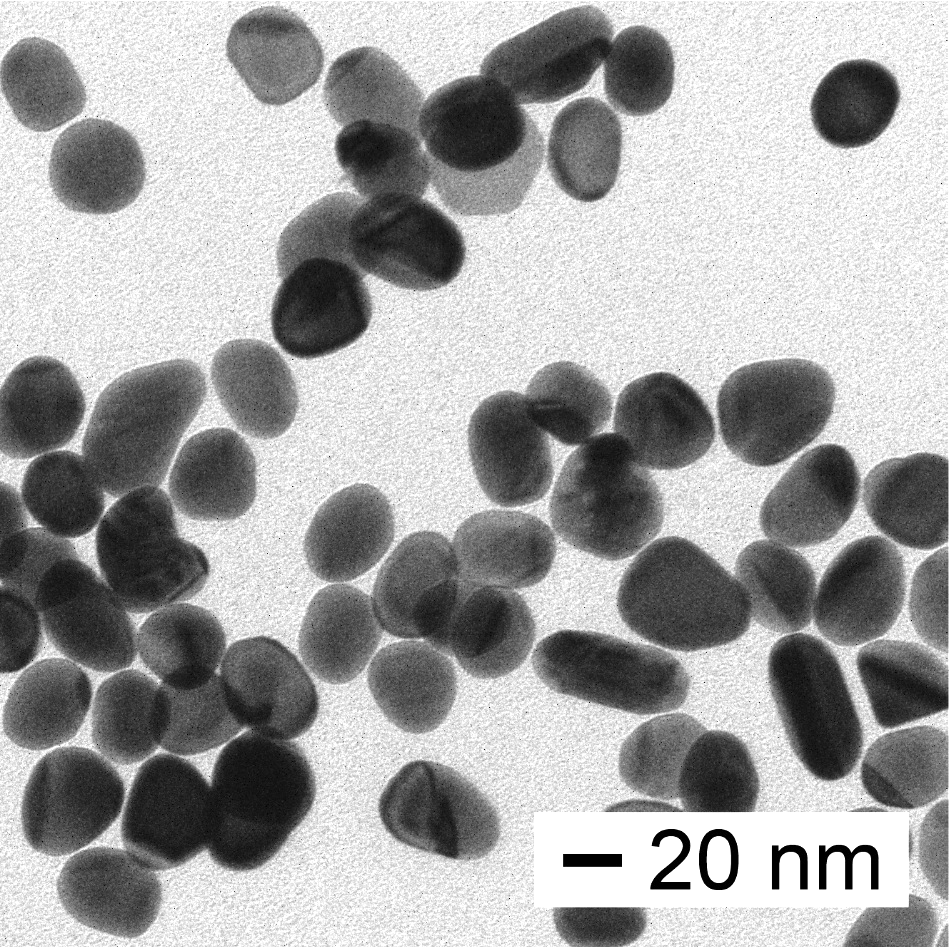


**Figure S2.** TEM image of the non-uniform Au nanoparticles synthesized using a one-shot injection method at a scale of 15 mg per day.


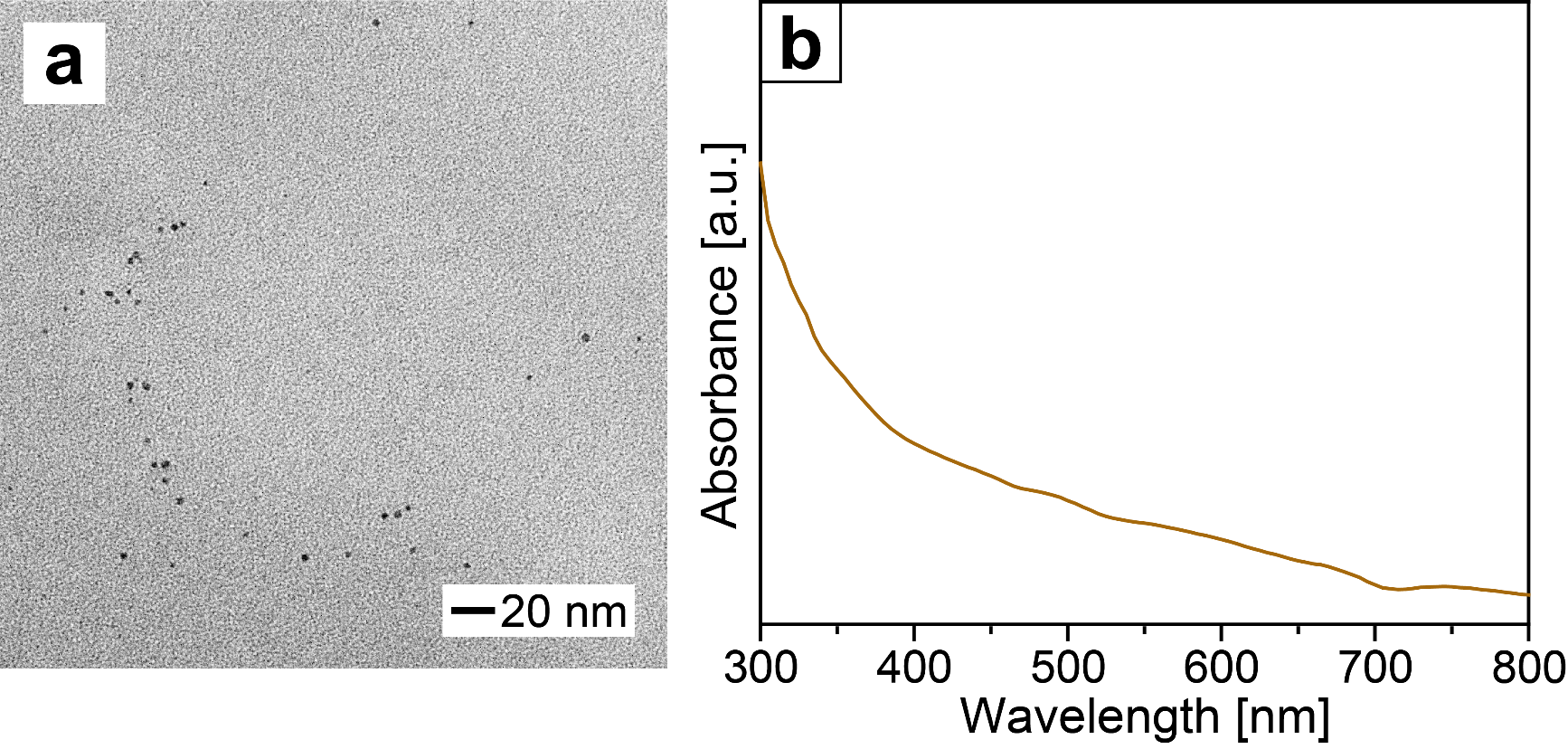


**Figure S3.** (a) TEM image and (b) UV-vis spectrum of an aqueous suspension of the initially formed Au clusters.


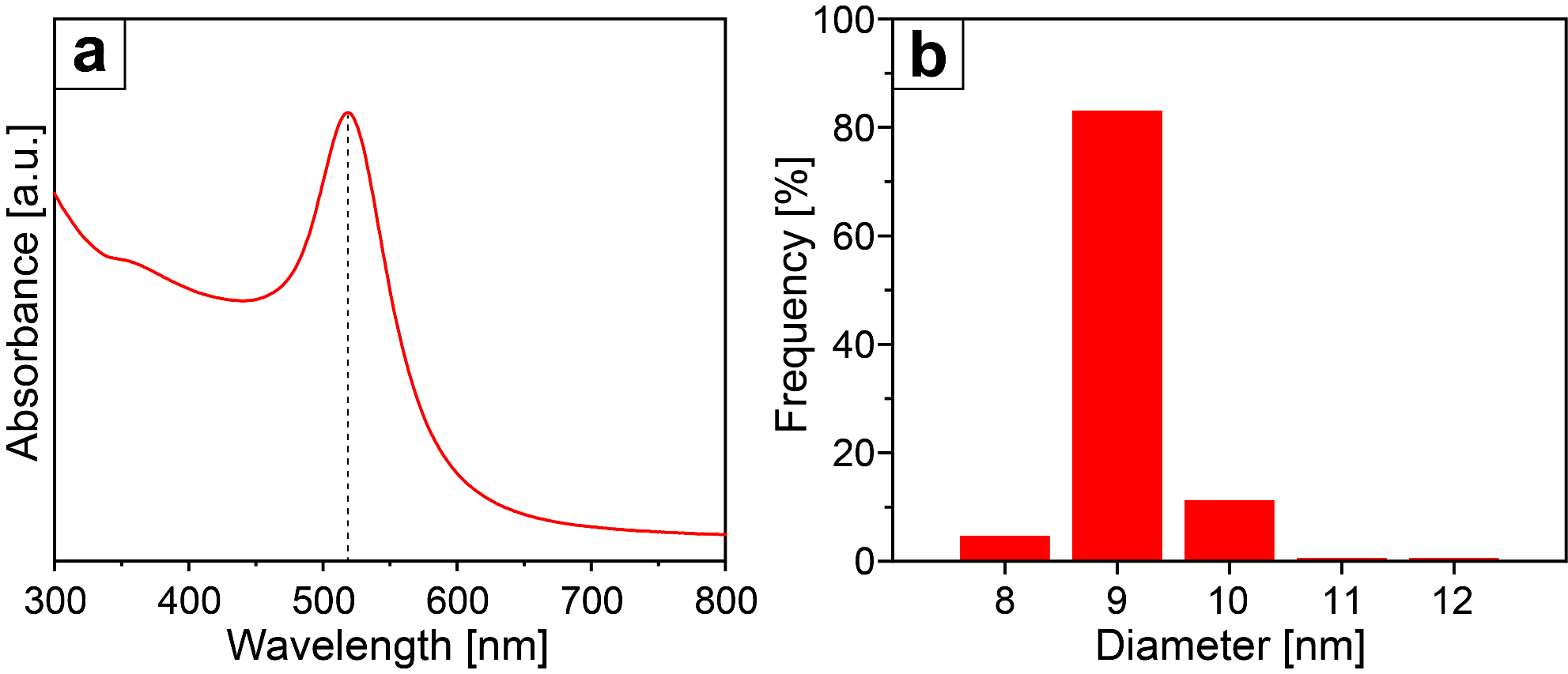


**Figure S4.** (a) UV-vis spectrum and (b) diameter distribution of the small Au spheres.


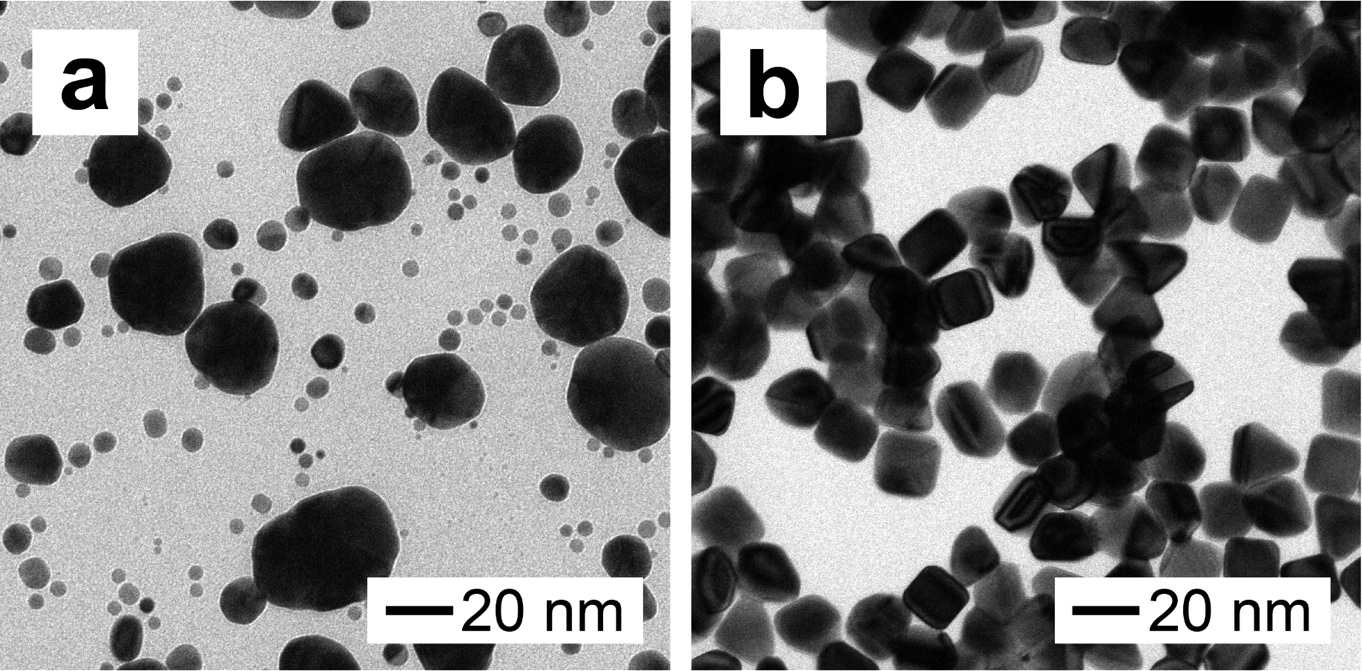


**Figure S5.** TEM images of the (a) small nanospheres and (b) nanocubes obtained under the condition of slow flow rate without a mixer.


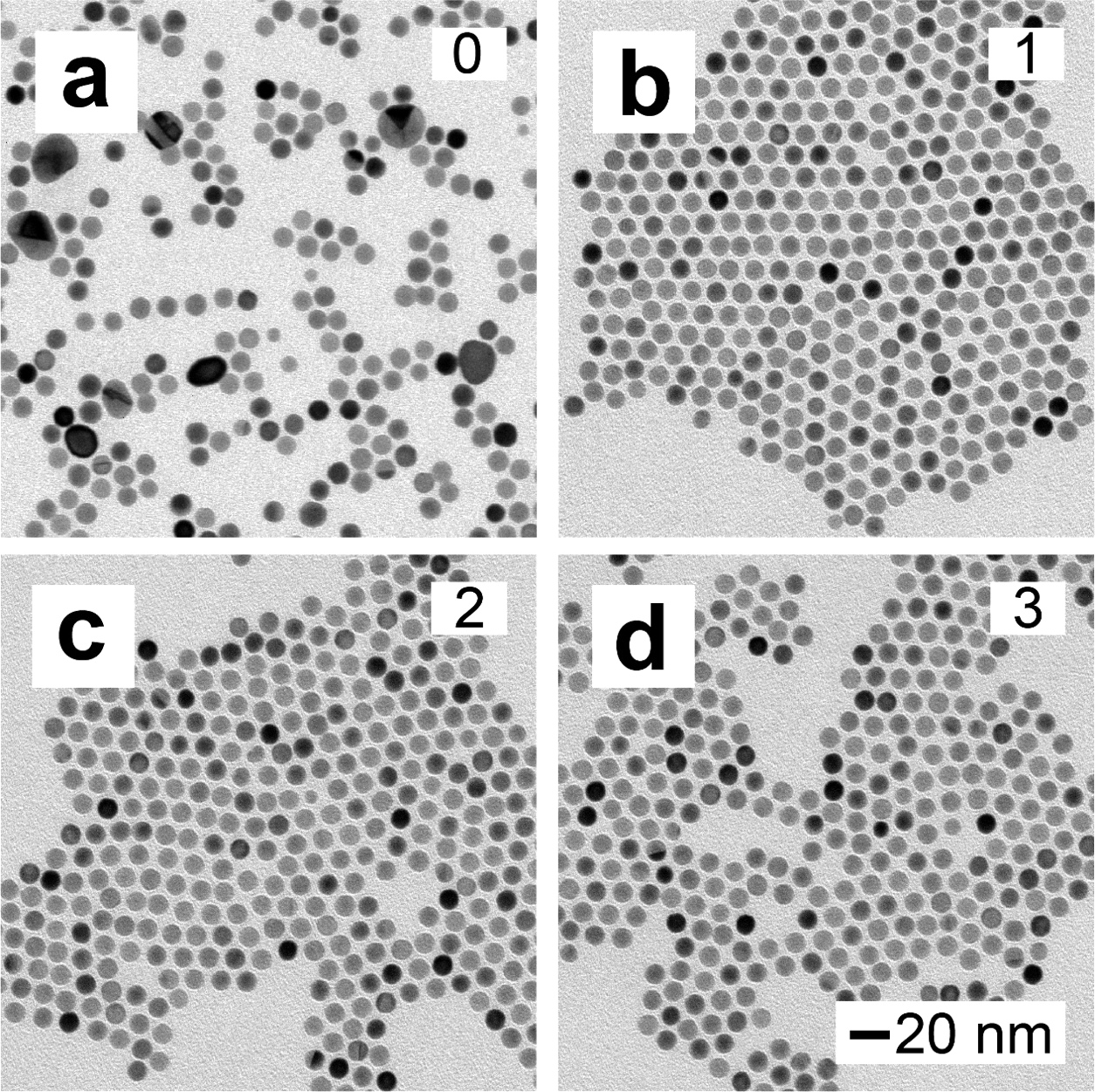


**Figure S6.** TEM images showing the small Au nanospheres synthesized using 0, 1, 2, and 3 mixers, respectively. The scale bar applies to all panels.


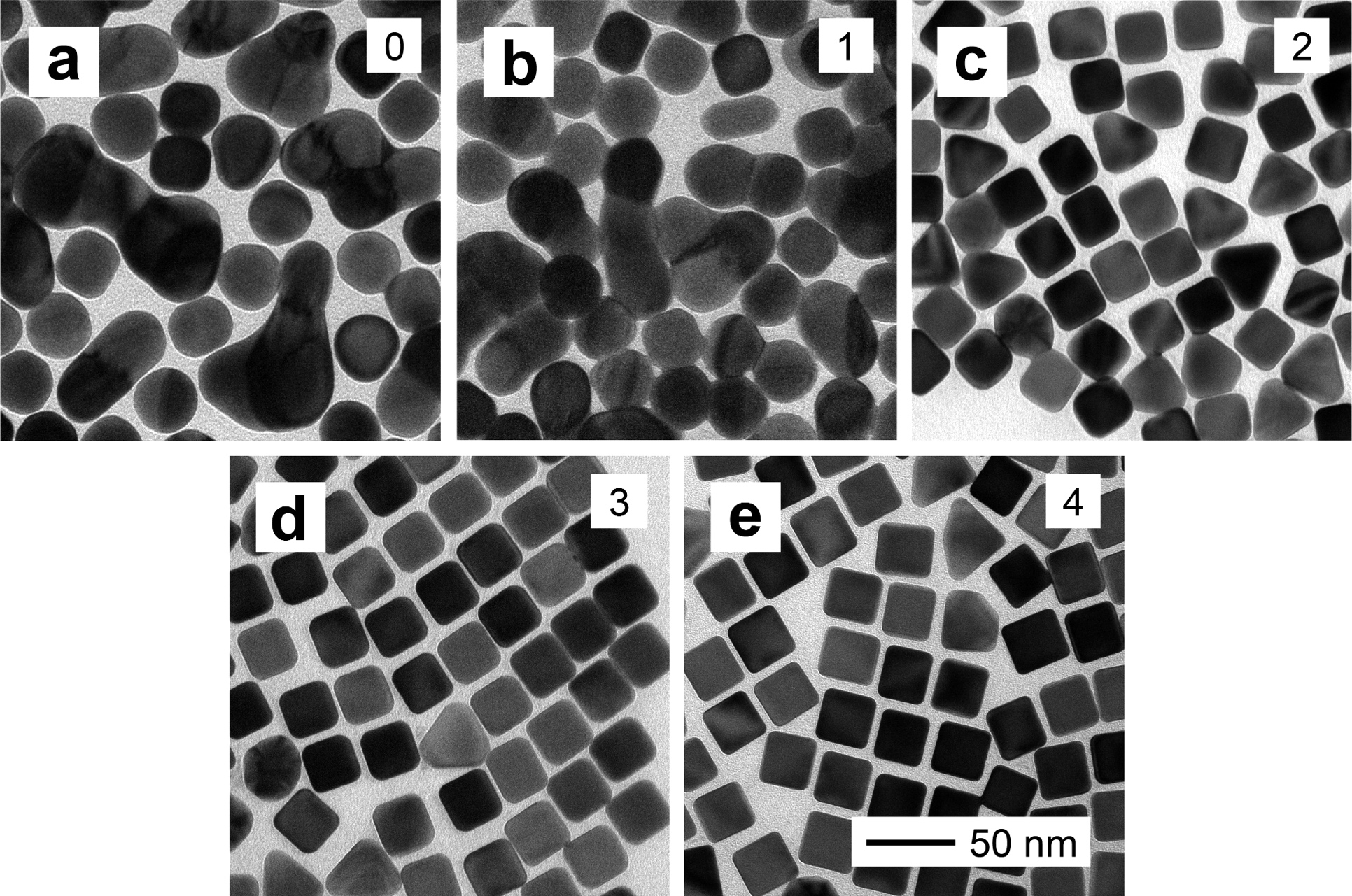


**Figure S7.** (a-e) TEM images showing the Au nanocubes synthesized using 0, 1, 2, 3, and 4 mixers, respectively. The scale bar applies to all panels.


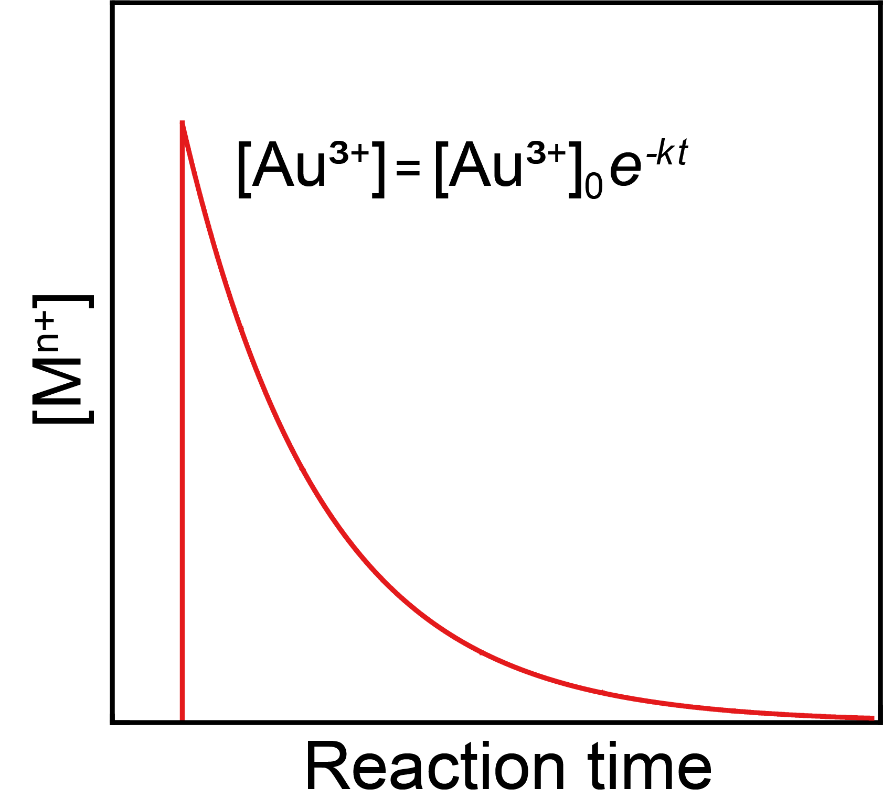


**Figure S8.** Simulated precursor concentration as a function of reaction time in the case of one-shot injection and a batch reactor.


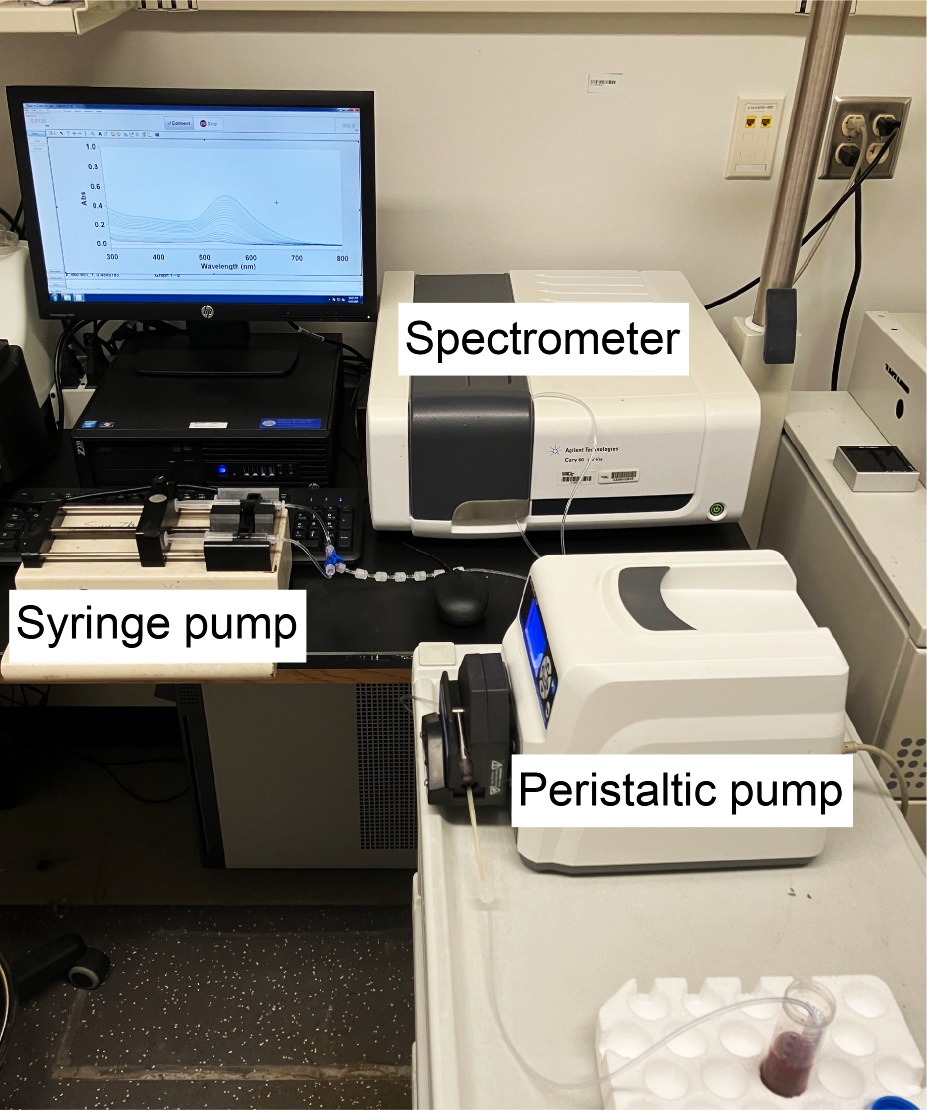


**Figure S9.** Digital photograph of the setup used for in situ UV–vis monitoring.


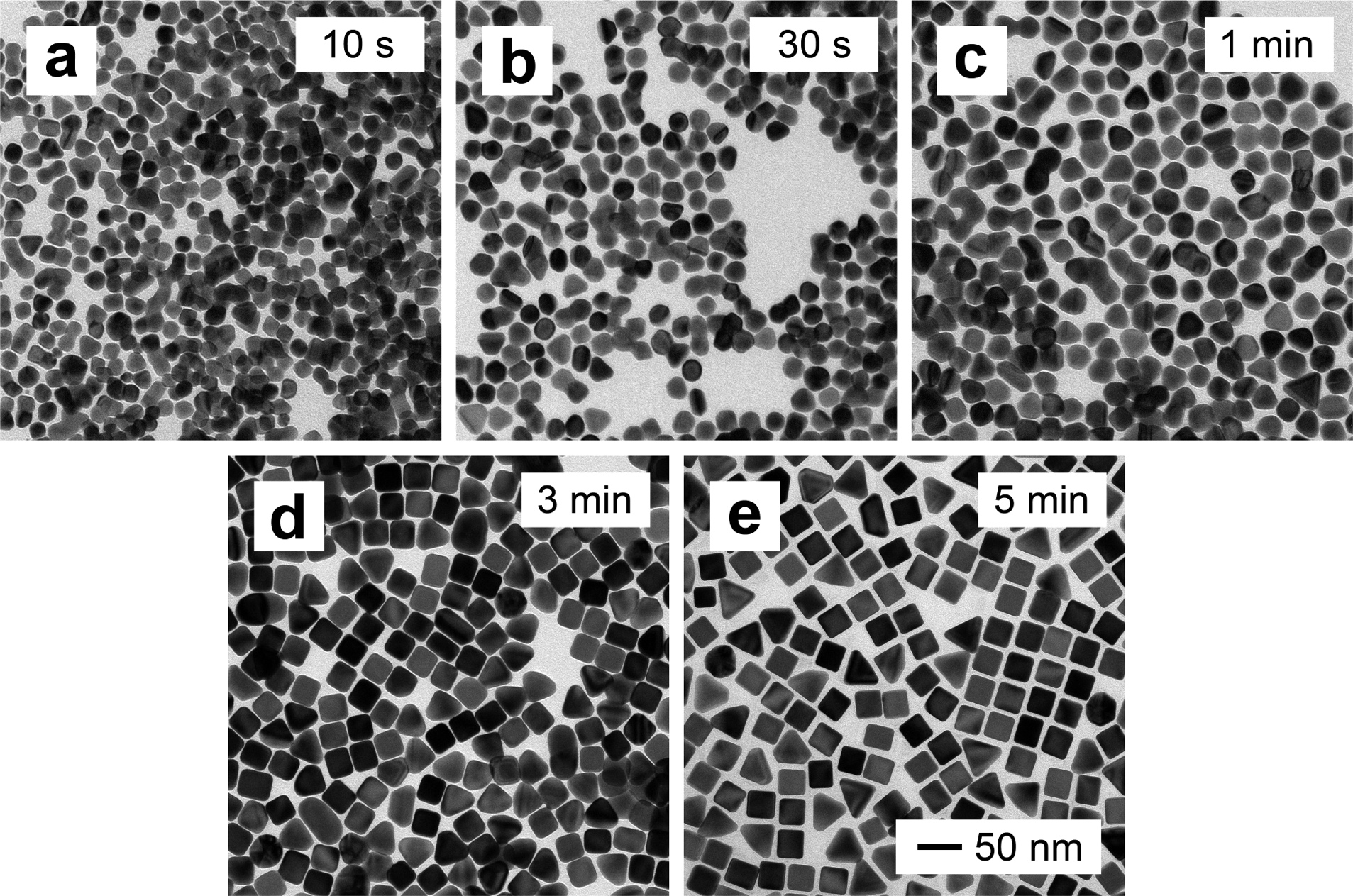


**Figure S10.** TEM images showing the Au nanocubes obtained at 10 s, 30 s, 1 min, 3 min, and 5 min, respectively, into the synthesis. The scale bar applies to all panels.


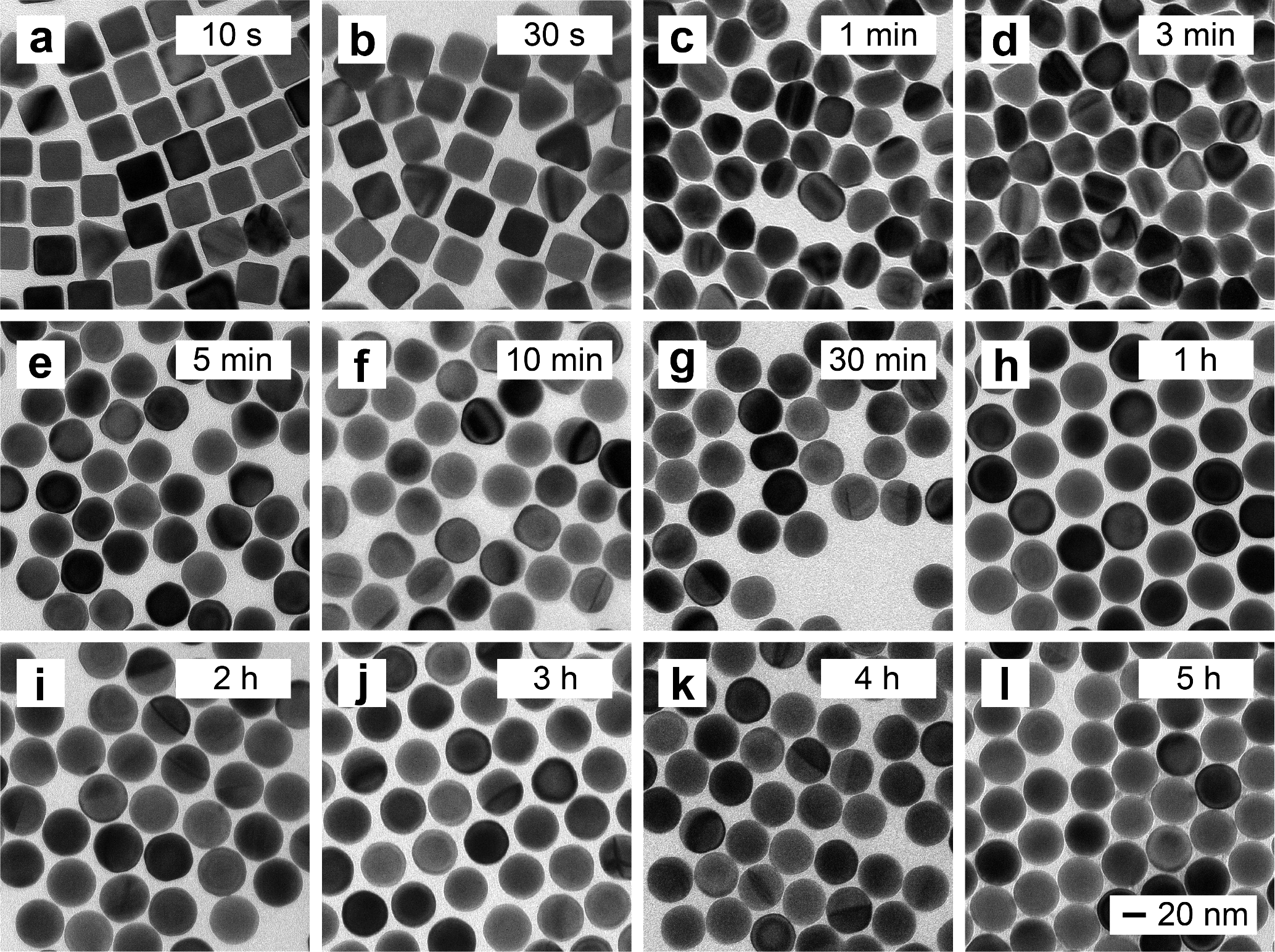


**Figure S11.** TEM images showing the large Au nanospheres obtained at 10 s, 30 s, 1 min, 3 min, 5 min, 10 min, 30 min, 1 h, 2 h, 3 h, 4 h, and 5 h, respectively, into the incubation. The scale bar applies to all panels.


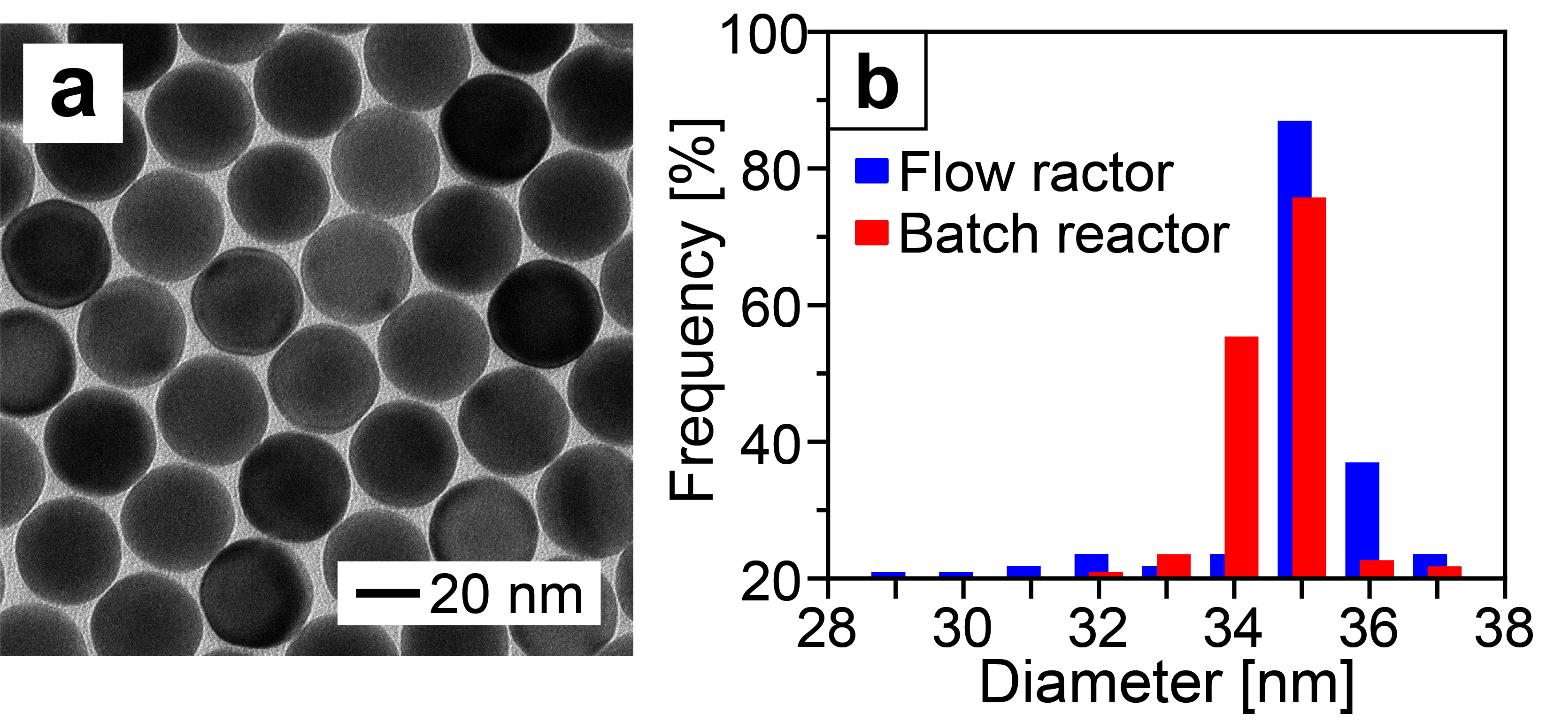


**Figure S12.** (a) TEM image of the 35-nm nanospheres obtained by incubation in a batch reactor. (b) Diameter distributions of the Au nanospheres obtained by incubation in a flow (blue) and a batch (red) reactor, respectively.


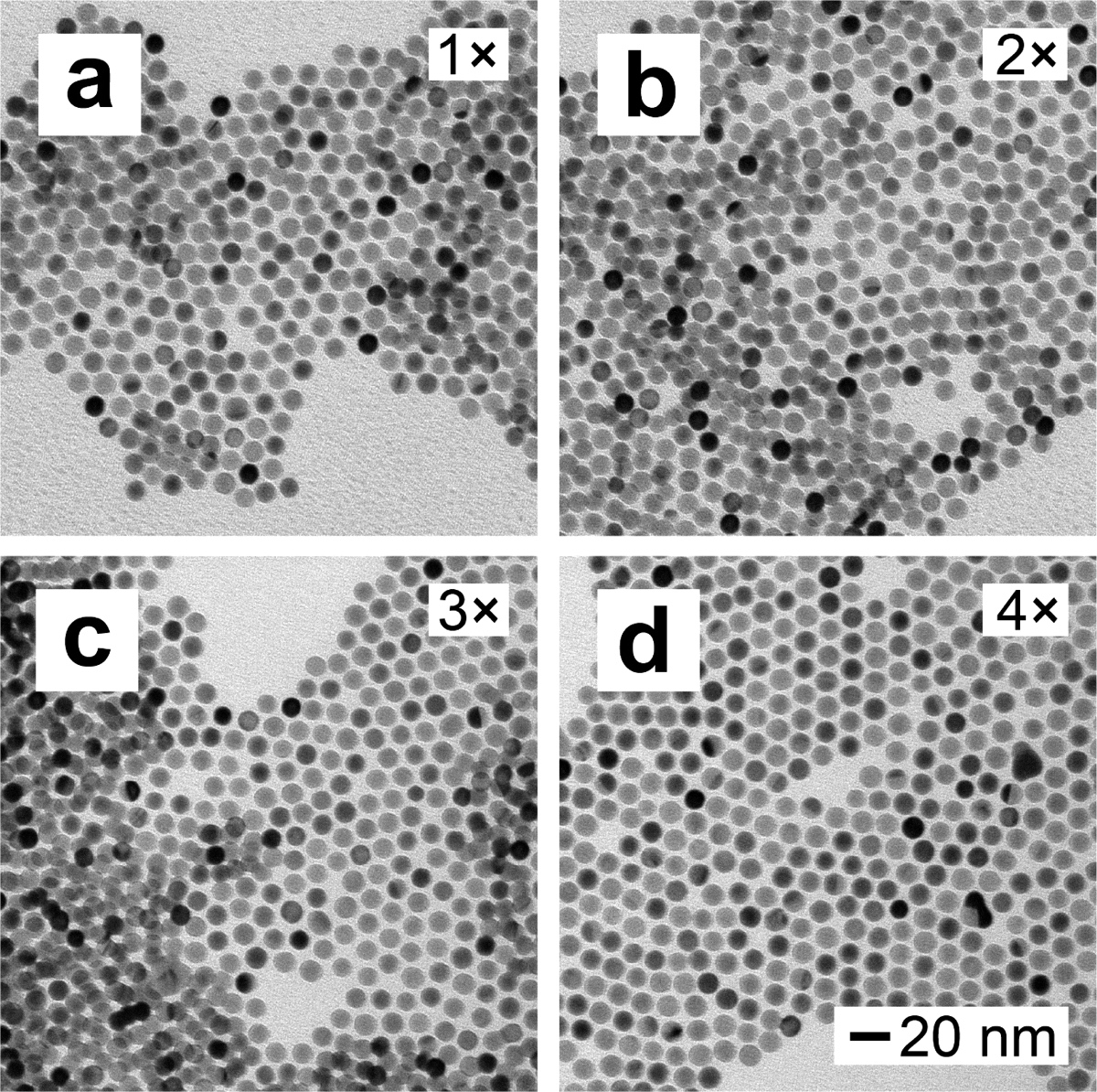


**Figure S13.** TEM images showing the 9-nm Au spheres obtained at 1×, 2×, 3×, and 4×, respectively, of the concentrations of both the seed and precursor used in a standard synthesis, while keeping other parameters the same. The scale bar applies to all panels.


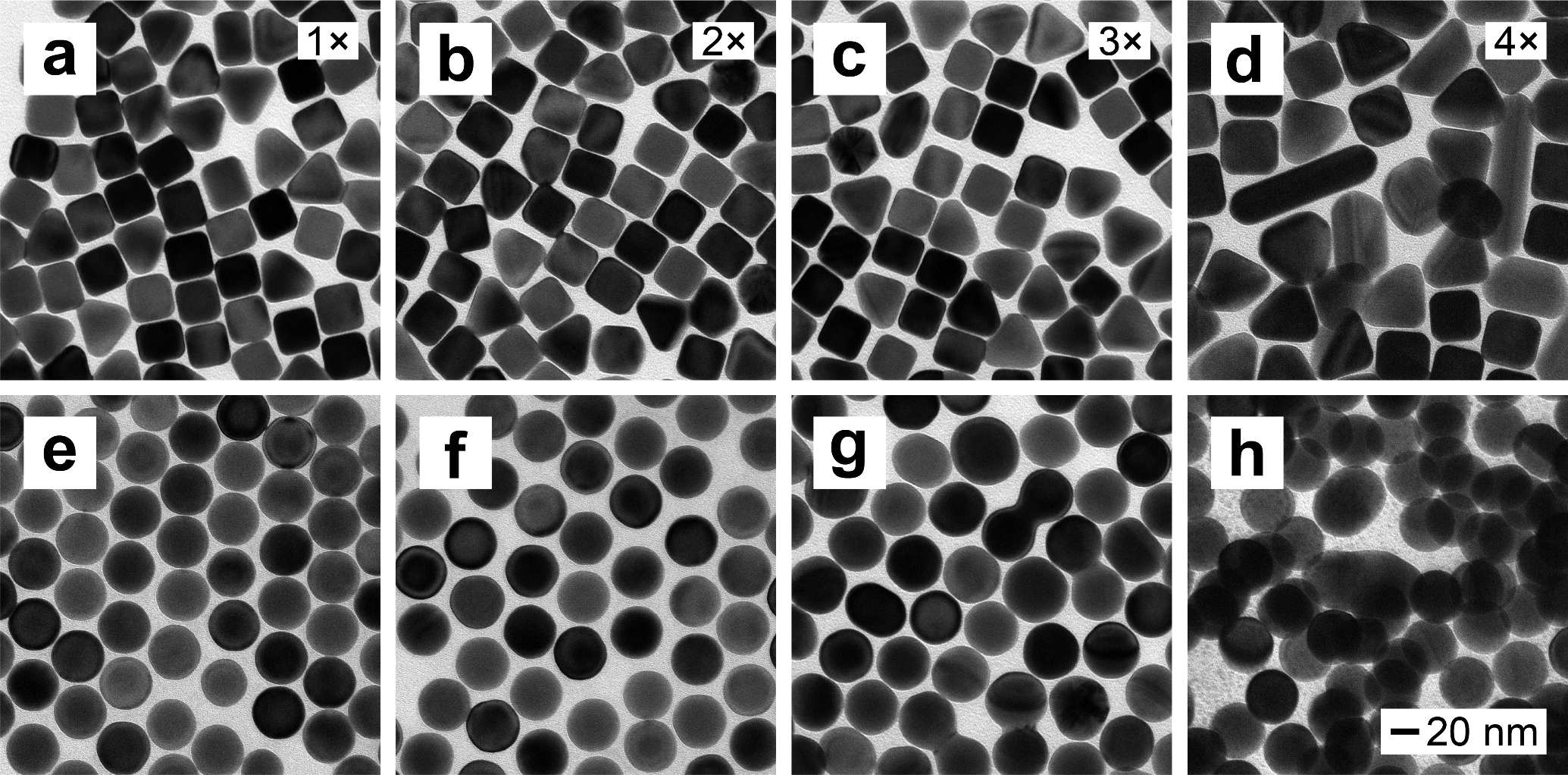


**Figure S14.** (a-d) TEM images showing the 30-nm Au cubes obtained at 1×, 2×, 3×, and 4×, respectively, the concentrations of all reactants while keeping other parameters the same. (e-h) TEM images of the large Au nanospheres obtained after incubating the samples shown in (a-d), respectively. The scale bar applies to all panels.


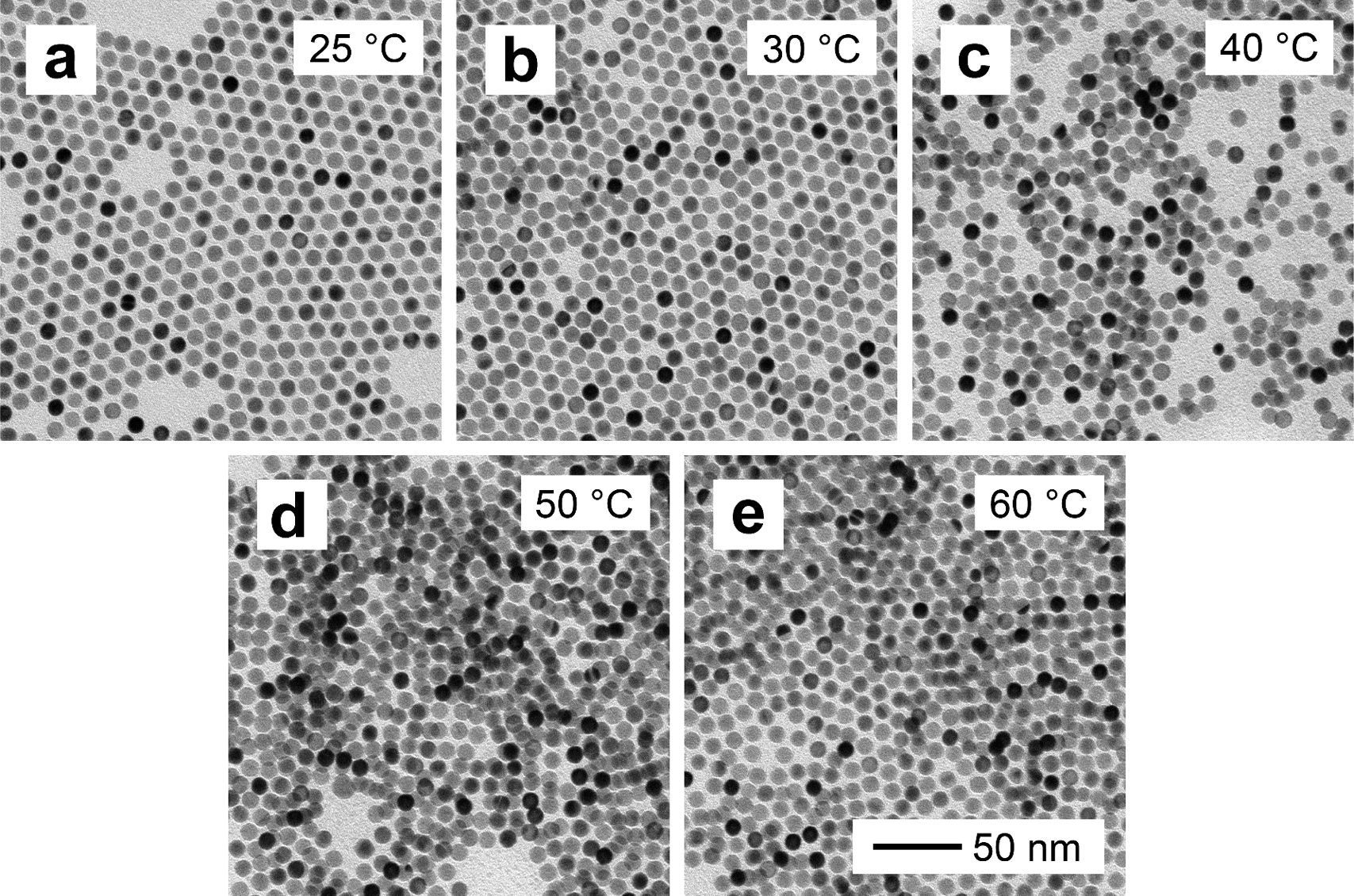


**Figure S15.** TEM images showing the 9-nm Au spheres obtained at 25, 30, 40, 50, and 60 °C, respectively, while keeping other parameters the same. The scale bar applies to all panels.


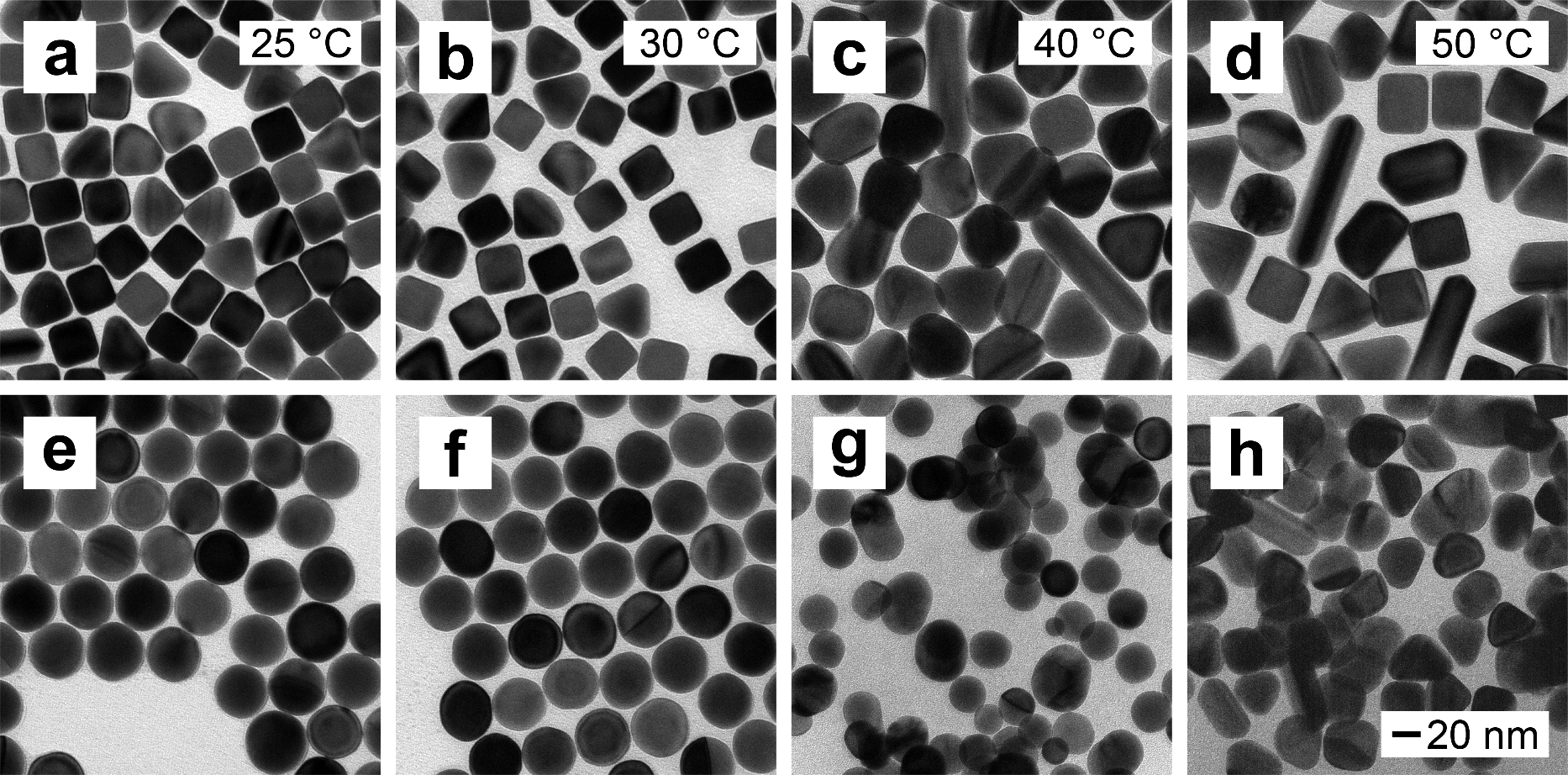


**Figure S16.** (a-d) TEM images showing the 30-nm Au cubes obtained at 25, 30, 40, and 50 °C, respectively, while keeping other parameters the same. (e-h) TEM images of the large Au nanospheres obtained after incubating the samples shown in (a-d), respectively. The scale bar applies to all panels.


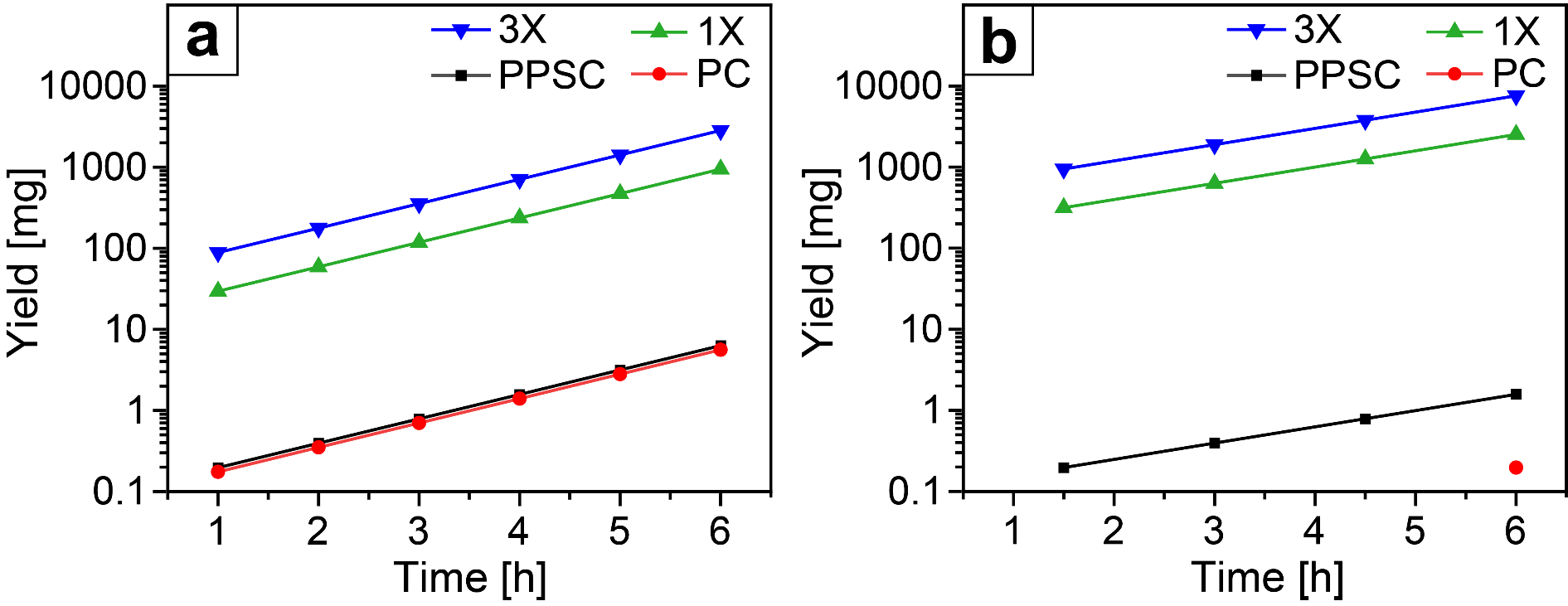


**Figure S17.** The yields for the synthesis of (a) 9-nm spheres and (b) 35-nm spheres using the protocol reported in this study (blue: three-fold concentration; green: one-fold concentration), compared with those from reference 1 (PPSC: the protocol reported in *Part. Part. Syst. Charact.*, black) and reference 2 (PC: the protocol reported in *Precis. Chem.*, red).


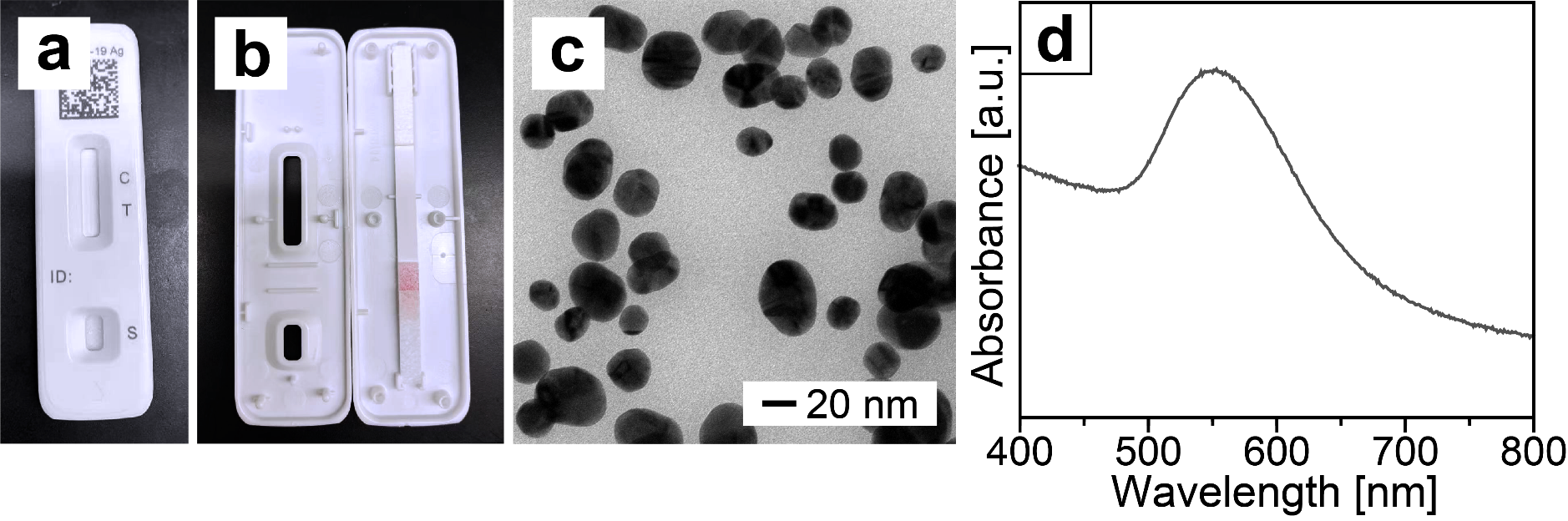


**Figure S18.** (a and b) Digital photographs of a commercial COVID-19 LFT kit. (c)TEM image of the Au nanoparticles harvested from the commercial LFT kit. (d) UV-vis spectrum of an aqueous suspension of the Au nanoparticles in the commercial COVID-19 LFT kits.


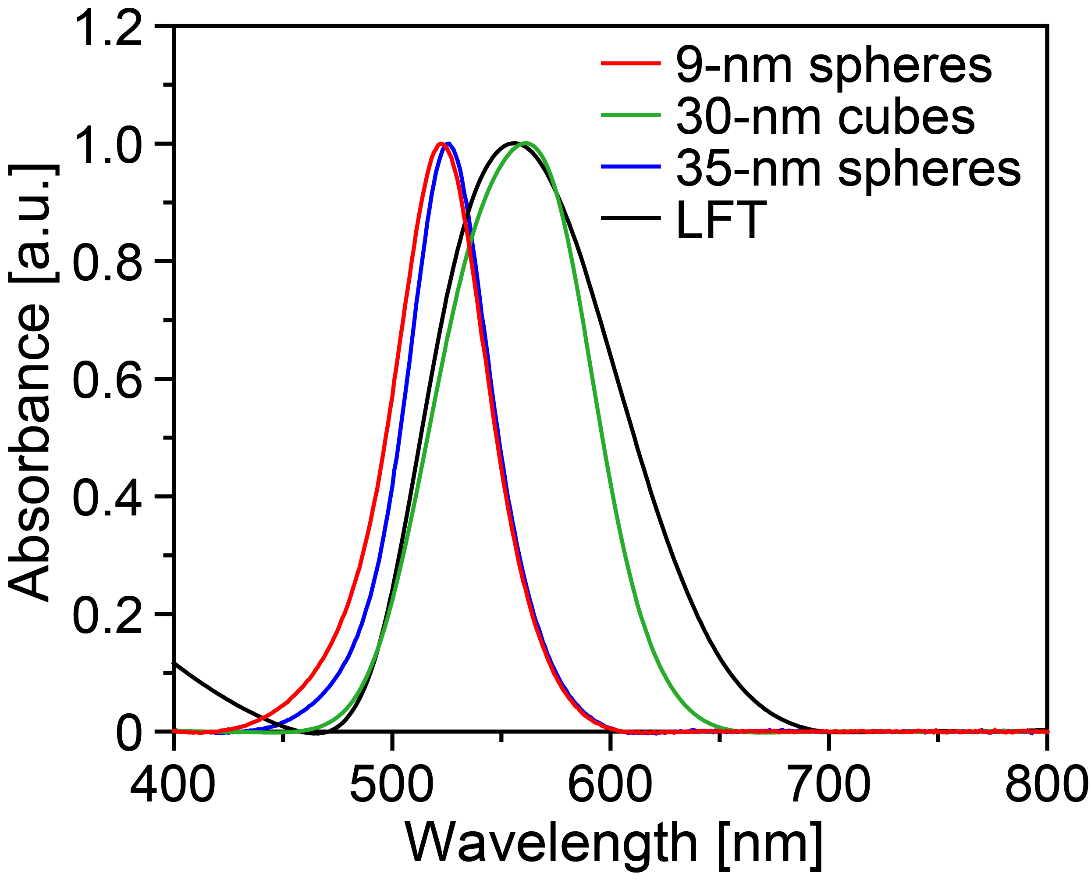


**Figure S19.** Baseline-subtracted and peak-normalized UV–vis spectra of aqueous suspensions of the four types of Au nanocrystals. Baselines were subtracted with identical parameters for all samples prior to normalization.


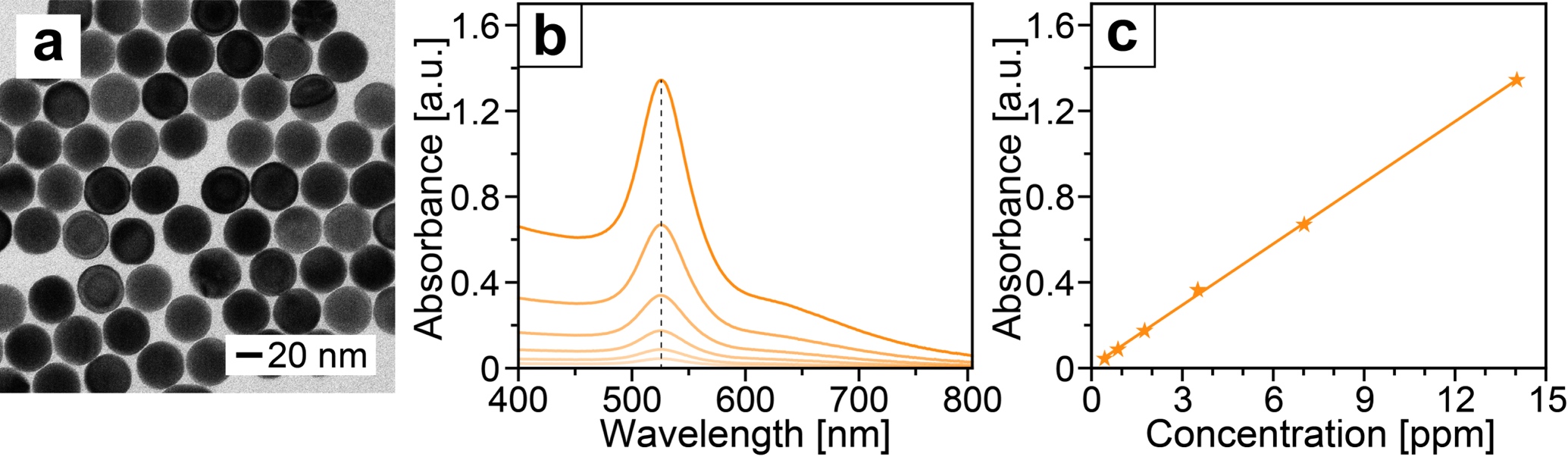


**Figure S20.** (a) TEM images of the 35-nm spheres covered by tri-citrate(3H^+^). (b) UV-vis spectra of aqueous suspensions of the 35-nm spheres after modification with tri-citrate(3H^+^), under progressively dilution (by a factor of two) as the color of the curve fades away. (c) Plots showing the relationship between the peak absorbance and the concentration of 35-nm spheres covered by tri-citrate(3H^+^).

**Table S1.** Summarized detailed reagent compositions of the standard synthesis method for Au nanocrystals in flow reactors.

| Products | Reagent A | Reagent B |
| --- | --- | --- |
| Cluster | CTAB (200 mM), HAuCl_4_ (0.5 mM) | NaBH_4_ (1.2 mM) |
| Small sphere | CTAC (73 mM), aqueous suspension of cluster (1.8% v/v) | CTAC (73 mM), HAuCl_4_ (0.36 mM), AA (55 mM) |
| Cube | CTAC (100 mM), AA (0.65 mM), KBr (0.625 mM), small sphere (1.6 μg mL^-1^) | HAuCl_4_ (0.5 mM) |
| Large sphere | Outlet of cube synthesis process | CTAC (756 mM) |

**Table S2.** Summarized detailed workflow of the standard synthesis method for Au nanocrystals in flow reactors.

| Products | Flow rate of reagent A  [mL min^-1^] | Flow rate of reagent B  [mL min^-1^] | Temperature  [°C] | Reaction time  [min] | Post reaction process |
| --- | --- | --- | --- | --- | --- |
| Cluster | 10 | 10 | 25 | 180 | - |
| Small sphere | 10 | 10 | 25 | 15 | Washed with H_2_O, re-dispersed into aqueous CTAC (20 mM) |
| Cube | 10 | 10 | 25 | 30 | Washed with H_2_O, re-dispersed into aqueous CTAC (20 mM) |
| Large sphere | 10 | 10 | 95 | 300 | Washed with H_2_O, re-dispersed into aqueous CTAC (20 mM) |

**References**

[1] Y. Zheng, X. Zhong, Z. Li, Y. Xia, *Part. Part. Syst. Charact.* **2014**, *31*, 266.

[2] K. K. Li, J. He, Q. Huang, S. Kinoshita, Y. Ding, Y. Xia, *Precis. Chem.* **2025**, *3*, 272.
